# Hemodynamics affects factor XI/XII anticoagulation efficacy in patient-derived left atrial models

**DOI:** 10.1016/j.cmpb.2025.108761

**Published:** 2025-04-21

**Authors:** M. Guerrero-Hurtado, M. García-Villalba, A. Gonzalo, E. Durán, P. Martinez-Legazpi, P. Ávila, A.M. Kahn, M.Y. Chen, E. McVeigh, J. Bermejo, J.C. del Álamo, O. Flores

**Affiliations:** a Department of Aerospace Engineering, Universidad Carlos III de Madrid, Leganés, Spain; b Institute of Fluid Mechanics and Heat Transfer, TU Wien, 1060 Vienna, Austria; c Department of Mechanical Engineering, University of Washington, Seattle, WA, USA; d Department of Mechanical, Thermal and Fluids Engineering, Universidad de Málaga, Málaga, Spain; e Dept. of Mathematical Physics and Fluids, Universidad Nacional de Educación a Distancia, Spain; f CIBERCV, Madrid, Spain; g Division of Cardiovascular Medicine, University of California San Diego, La Jolla, CA, USA; h National Heart, Lung, and Blood Institute, National Institutes of Health, Bethesda, MD, USA; i Department of Bioengineering, University of California San Diego, La Jolla, CA, USA; j Department of Radiology, University of California San Diego, La Jolla, CA, USA; k Hospital General Universitario Gregorio Marañón, Madrid, Spain; l Instituto de Investigación Sanitaria Gregorio Marañón, Madrid, Spain; m Facultad de Medicina, Universidad Complutense de Madrid, Madrid, Spain; n Center for Cardiovascular Biology, University of Washington, Seattle, WA, USA; o Division of Cardiology, University of Washington, Seattle, WA, USA

**Keywords:** Digital twins, Coagulation cascade, Factor XI, Factor XII, Computational fluid dynamics

## Abstract

**Background and Objective:**

Atrial fibrillation (AF) is a common arrhythmia that disrupts blood circulation in the left atrium (LA), causing stasis in the left atrial appendage (LAA) and increasing thromboembolic risk. In patients at sufficiently high risk, anticoagulation is indicated. This benefit may be counterbalanced by an increased risk of bleeding. Novel anticoagulants under development, such as factor XI/XII inhibitors, may be associated with a lower bleeding risk. However, their efficacy in preventing thrombosis is not fully understood. We hypothesized that patient-specific flow patterns in the LA and LAA not only influence the risk of thrombosis but also the effectiveness of anticoagulation agents.

**Methods:**

To test our hypothesis, we simulated blood flow and the intrinsic coagulation pathway in patient-specific LA anatomies with and without factor XI/XII inhibition. We included a heterogeneous cohort of thirteen patients, some in sinus rhythm and others in AF, four of whom had an LAA thrombus or a history of transient ischemic attacks. We used computational fluid dynamics based on 4D CT imaging and a detailed 32-coagulation factor system to run 247 simulations. We analyzed baseline LA flow patterns and evaluated various factor XI/XII inhibition levels. Implementing a novel multi-fidelity coagulation modeling approach accelerated computations by two orders of magnitude, enabling many simulations to be performed.

**Results:**

The simulations provided spatiotemporally resolved maps of thrombin concentration throughout the LA, showing that it peaks inside the LAA. Coagulation metrics based on peak LAA thrombin dynamics suggested patients could be classified as having no, moderate or high thromboembolic risk. High-risk patients had slower flows and higher residence times in the LAA than those with moderate thromboembolic risk, and they required stronger factor XI/XII inhibition to prevent thrombin growth. These data suggest that the anticoagulation effect was also related to the LAA hemodynamics.

**Conclusion:**

The methodology outlined in this study has the potential to enable personalized assessments of coagulation risk and to tailor anticoagulation therapy by analyzing flow dynamics in patient-derived LA models, representing a significant step towards advancing the application of digital twins in cardiovascular medicine.

## 1. Introduction

Atrial fibrillation (AF) is a common arrhythmia affecting between 20% and 33% of individuals older than 45 during their lifetime [1]. In AF, the cyclic contraction of the atria is replaced by a rapid yet erratic and weak trembling motion that disturbs blood flow. AF-associated flow is particularly aberrant inside the left atrial appendage (LAA), a small, narrow sac protruding the left atrium (LA), where thrombosis is most likely [2].

Patients with AF have increased risk of dementia, heart failure, and death, in many cases associated with embolic events triggered by left atrial thrombosis. Therefore, preventing thromboembolism is crucial in the management of AF. Oral anticoagulation in AF is recommended only when the annual stroke risk exceeds approximately 1% [3]. However, current clinical tools used for risk stratification – primarily the CHA2DS2-VASc score – fail to differentiate patients with intermediate risks and do not consider known additional modifiers such as the AF pattern, LAA velocities, spontaneous contrast in the LA, or comorbidities such as cancer and chronic kidney disease [4]. Furthermore, the benefits of oral anticoagulation need to be carefully balanced against the risk of bleeding, which frequently leads to the discontinuation of therapy or the need to reduce the drug dose [5].

Direct oral anticoagulants (DOACs) are the therapy of choice for preventing stroke in AF. Compared to traditional vitamin K antagonists, DOACs reduce the risk of systemic embolism and intracranial bleeding, with added advantages of lower monitoring needs and fewer food and drug interactions [5,6]. Except for dabigatran, a direct thrombin inhibitor, current DOACs target thrombin amplification by reducing the concentration of activated factor X [7]. Factor Xa plays a critical function in coagulation, connecting the extrinsic pathway, externally triggered by vascular injury, with the self-initiated intrinsic pathway [8].

The efficacy and dose–response curves for DOACs have never been established for the relevant clinical target of preventing LA thrombosis. Current dosing schemes of DOACs are based on their individual pharmacokinetic profiles, grounded on factors that condition the level of bioavailability such as age, genetics, renal function and metabolism [9–13]. However, despite correct dosing and treatment compliance, thromboembolic events are still more frequent in patients with AF than in the global population, suggesting that current DOAC prescriptions may not be adequate for certain high-risk patients [5]. Consequently, the field of novel DOAC drugs is an important area of current research, with new drugs being developed to target the inhibition of various coagulation factors [14]. Intrinsic pathway activation, mediated by factor XI/XII, has been identified as a therapeutic target with potential to reduce bleeding risk [15]. Factor XI antagonists have been proven significantly safer than currently used DOACs [16] but their relative efficacy is still uncertain [17,18]. Overall, the increasing availability of drugs targeting different coagulation factors and their dose dependence create a need for improved models to understand their mechanism of action [19].

The idea that flow patterns affect thrombosis is universally accepted as a pillar of the Virchow’s triad [20]. Therefore, it seems plausible that the effects of inhibiting coagulation factors will also be sensitive to patient-specific flow patterns. Coagulation tests measure the kinetics of relevant coagulation cascade species using laboratory test kits that do not reproduce flow conditions, let alone patient-specific ones. Therefore, the accuracy of these tests’ results may thus vary from patient to patient.

Coagulation under flow can be modeled mathematically by a system of coupled advection–diffusion–reaction (ADR) partial differential equations (PDEs), one for each reacting component [21]. Traditionally, these equations are solved numerically together with the Navier–Stokes equations governing blood flow using computational fluid dynamics (CFD). However, this approach is challenging due to the large number of components involved, the fine 3D meshes required to resolve their concentration gradients, and the disparate timescales governing coagulation [22]. To the best of our knowledge, no comprehensive CFD studies have addressed intracardiac coagulation across various clinical scenarios or evaluating individual responses to different levels of anticoagulation.

**Fig. 1.**
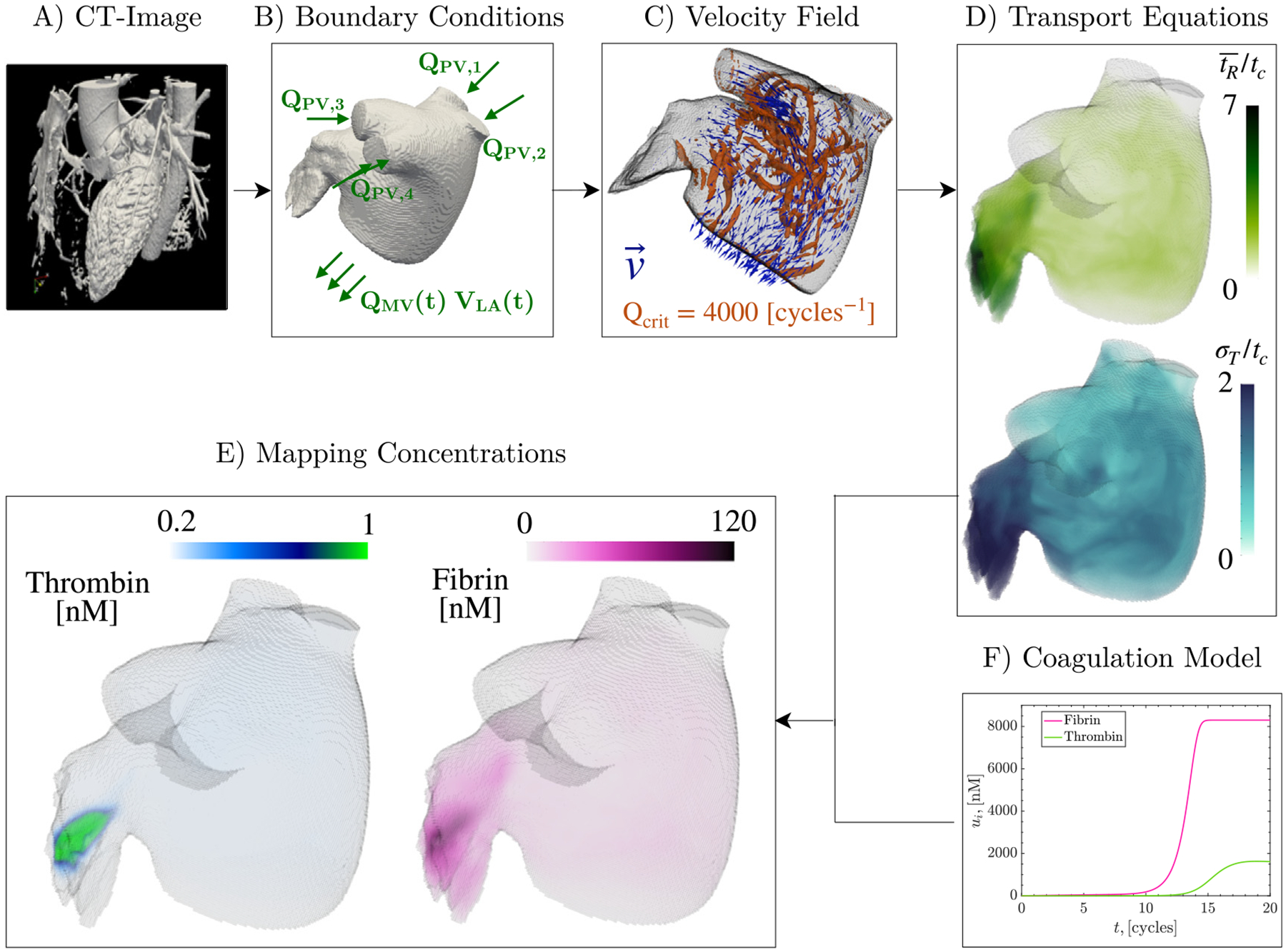
Workflow for Multi-fidelity approach of the coagulation cascade in patient-specific anatomies: The LA wall motion is obtained from CT imaging. Subsequently, the total flow rate through the PVs ($Q_{PV}$) is calculated from mass conservation in the LA volume and evenly distributed through each PV ($Q_{PV,i}$). The velocity field ($\vec{v}$), residence time $\bar{t_{R}}$, and its higher order moments (e.g., $\bar{t_{R}^{2}}$) are computed by solving the Navier–Stokes equations for incompressible flow using computational fluid dynamics and transport Eqs. (3)–(4). A 32-species ODE coagulation model is solved for different levels of factor XI/XII inhibition, and each species’ spatial concentration field is mapped using multi-fidelity (MuFi) modeling (Eqs. (6)–(7)).

We present an extensive set of simulations examining flow, coagulation in the LA, and the role of inhibition of factors XI and XII using a set of patient-specific 3D anatomies. The workflow employed to perform these simulations is summarized in Fig. 1. We combined CFD analysis with a novel multi-fidelity (MuFi) coagulation model [23], which decouples the blood flow and coagulation solvers, accelerating simulations by two orders of magnitude. In previous works, the MuFi approach was tested in simplified geometries. In this work, we validated the MuFi approach for the first time in realistic patient derived LA geometries. We analyzed the data from 13 patients including cases in sinus rhythm and AF, patients with and without thromboembolic events (LAA thrombus or cerebrovascular accidents), and different anticoagulation regimens. Overall, we performed 247 simulations considering 32 coagulation factors and 19 levels of factor XI/XII inhibition per patient. Thrombin levels were highest in the LAA of patients with poor blood washout, and the effectiveness of new anticoagulants targeting the intrinsic coagulation pathway was also worse in these patients. The new computational tools introduced in this manuscript could open new venues for improving thromboembolic risk stratification and tailoring anticoagulant prescription in AF patients.

The manuscript is organized as follows. The methodology is presented in Section 2, including a brief description of the MuFi model, the coagulation cascade kinetics used in the models, and the patient-derived CFD simulations. Section 3 includes the verification of the MuFi models for patient-derived LA flow, the description of the thrombin concentrations without inhibition, and the effects of factors XI/XII inhibition. Discussion and conclusions are provided in Sections 4 and 5, respectively.

## 2. Methods

### 2.1. High-fidelity (HiFi) and multi-fidelity (MuFi) models of the coagulation cascade under flow

Considering blood as a continuum flowing with velocity $\vec{v}(\vec{x},t)$, the evolution of the concentration of coagulation components (factors, regulatory proteins, enzymes and other substances) is modeled by a system of advection–diffusion–reaction equations:

(1) $$\frac{\partial u_{i}}{\partial t}+\nabla \cdot \left(\vec{v}u_{i}\right)=R_{i}+D_{i}\nabla^{2}u_{i},\;\text{for}\;i=1,\ldots ,N,$$

where $u_{i}(\vec{x},t)$ for i=1…N are concentration fields of the N components involved. The terms $R_{i}\left(u_{1},u_{2},\ldots ,u_{N}\right)$ denote the reaction rates from chemical kinetics, and $D_{i}$ stand for their diffusivity coefficients. We refer to this system of N partial differential equations (PDEs) as the high-fidelity (HiFi) model. Given knowledge of $\vec{v}(\vec{x},t)$, this HiFi model can be solved with appropriate initial and boundary conditions for $u_{i}$. Dirichlet boundary conditions are enforced at the flow inlets (i.e., on the pulmonary veins for simulations of the LA flow) as $u_{i}=u_{i,0}$, while homogeneous Neumann boundary conditions ($\partial u_{i}/\partial n=0$) are applied at solid surfaces and flow outlets.

We can non-dimensionalize Eqs. (1) using the flow velocity scale $U_{c}$ and vessel length scale $L_{c}$:

(2) $$\frac{\partial u_{i}}{\partial \tau }+\nabla \cdot \left(\vec{v}u_{i}\right)=Da{\tilde{R}}_{i}+\frac{1}{Pe}\nabla^{2}u_{i},\;\text{for}\;i=1,\ldots ,N,$$

where $\tau =tU_{c}/L_{c}$ is a dimensionless time variable, ${\tilde{R}}_{i}=t_{r}R_{i}$ is a dimensionless reaction rate normalized with the characteristic time of the coagulation cascade $t_{r}$, the Damköhler number $Da=L_{c}/\left(t_{r}U_{c}\right)$ measures the relative importance of reaction kinetics and convective terms, and the Péclet number $Pe=U_{c}L_{c}/D_{i}$ measures the relative importance of convection over diffusion. Using typical values corresponding to the left atrium and the reaction rate and diffusivity of coagulation components (i.e., $U_{c}\sim 10\;\text{cm}/\text{s},L_{c}\sim 1\;\text{cm},t_{r}\sim 10^{2}\;\text{s},D_{i}\sim 10^{-6}{\;\text{cm}}^{2}/\text{s}$) yields $Da\sim 10^{-3}$ and $Pe\sim 10^{7}$. With a cardiac cycle period of $t_{c}=1\;\text{s}$, equivalent to $t_{c}=10L_{c}/U_{c}$, solving the HiFi model implies discretizing the domain into extremely fine grids due to the very large Péclet number, and running it for tens of cardiac cycles due to the Damköhler number of the reaction. Additionally, a complete description of the coagulation cascade typically involves dozens of coagulation components (N~50), leading to a large number of PDEs. Furthermore, many practical applications require multiple simulations sweeping over one or more parameters (i.e., initial and/or inlet concentrations of blood clotting factors, kinetic reaction constants, etc.), significantly increasing the compute time.

To improve the computational tractability of coagulation cascade modeling, we employed the Multi-Fidelity (MuFi) approach proposed by Guerrero-Hurtado et al. [23]. This method transforms the N advection–diffusion–reaction PDEs for the concentrations of the components into a set of ODEs using the blood residence time $\left(\bar{t_{R}}\right)$ as the independent variable. The resulting MuFi models require solving a single PDE for $\bar{t_{R}}$, integrating N ODEs for the concentrations of the components, and mapping the concentration fields as a function of the residence time, $u_{i}\left(\bar{t_{R}}(\vec{x},t)\right)$. This transformation is exact in the limit of zero diffusivity. For small but finite diffusivity, one can Taylor-expand $u_{i}(t)$ to include higher-order statistical moments of the residence time, such as $\bar{t_{R}^{2}},\bar{t_{R}^{3}},\ldots \bar{t_{R}^{p}}$. This expansion allows for deriving higher-order MuFi models that trade computational cost for order of accuracy.

In this study we employed three different MuFi models, with orders 1, 2 and 3, respectively. Depending on the order of the model, one or more of the following evolution equations are solved

(3) $$\frac{\partial \bar{t_{R}}}{\partial t}+\nabla \cdot \left(\vec{v}\bar{t_{R}}\right)=1,$$

(4) $$\frac{\partial \bar{t_{R}^{2}}}{\partial t}+\nabla \cdot \left(\vec{v}\bar{t_{R}^{2}}\right)=2\bar{t_{R}},$$

(5) $$\frac{\partial \bar{t_{R}^{3}}}{\partial t}+\nabla \cdot \left(\vec{v}\bar{t_{R}^{3}}\right)=3\bar{t_{R}^{2}},$$

as shown in Fig. 1D. As discussed in Section 2.5, these equations are numerically solved using a WENO scheme that introduces some numerical dissipation [23], not explicitly shown in the equations. After solving these PDEs, one can map the concentrations (Fig. 1E) for each MuFi model using the corresponding Taylor expansion

(6) $$u_{i}^{\text{MuFi}-1}=g_{i}\left(\bar{t_{R}}\right),$$

(7) $$u_{i}^{\text{MuFi}-2}=g_{i}\left(\bar{t_{R}}\right)+g^{\prime \prime }\left(\bar{t_{R}}\right)\frac{\sigma_{T}^{2}}{2},$$

(8) $$u_{i}^{\text{MuFi}-3}=g_{i}\left(\bar{t_{R}}\right)+g^{\prime \prime }\left(\bar{t_{R}}\right)\frac{\sigma_{T}^{2}}{2!}+g^{\prime \prime \prime }\left(\bar{t_{R}}\right)\frac{\gamma_{T}}{3!},$$

where the superindex indicate the order of the MuFi model, and the variables, $\sigma_{T}^{2}=\bar{t_{R}^{2}}-{\bar{t_{R}}}^{2}$ and $\gamma_{T}=\bar{t_{R}^{3}}-3\sigma_{T}^{2}\bar{t_{R}}-{\bar{t_{R}}}^{3}$ are the second- and third-order moments of the residence time centered in the mean. In these equations, $g_{i},g_{i}^{\prime \prime }$, and $g_{i}^{\prime \prime \prime }$ represent the solution and time derivatives of the concentration of the component i, determined by solving a system of N ODEs governing the dynamics of a well-mixed fluid volume with homogeneous initial conditions $\left(g_{i}(\vec{x},t=0)=u_{i}^{0}\right)$:

(9) $$\frac{dg_{i}}{d\bar{t_{R}}}=R_{i}\left(g_{1},g_{2},\ldots ,g_{N}\right)\;\text{for}\;i=1,\ldots ,N.$$

We refer to Eq. (9) as the no-flow reaction model. For the results presented in Section 3, the no-flow reaction model was integrated in time using an explicit, low-storage, 3-stage Runge–Kutta scheme. Once residence time and its higher-order moments are computed, the MuFi model allows for evaluating the coagulation cascade under multiple conditions by integrating the no-flow ODE system at almost negligible cost.

### 2.2. Factor XI/XII anticoagulant simulations: reaction kinetics and coagulation metrics

We implemented a system for 32 coagulation components with the reaction kinetics described by Zhu [24] in our MuFi model of factor XI/XII anticoagulants. This system is an adaptation of the system proposed by Kogan et al. [25], and it includes the detailed activation of factors XI and XII (necessary to assess the effect of anticoagulation therapies targeting the activation of the intrinsic pathway) and the reactions leading to the subsequent activation of factor X. We defined a single prothrombotic initial condition for all patients, with the concentration values reported in Table 1. All active factor concentrations were set to zero, except for thrombin (IIa) and factor XIIa. The factor XIIa concentration was selected from baseline values in previous studies [24,25]. The initial thrombin concentration was chosen within the high-end of the physiological range, to ensure that this species reached its maximum concentration within the simulated time of 20 cardiac cycles in the no-flow reaction model (Fig. 1F). This time frame is in accordance with activated partial thromboplastin time (aPTT), thrombin time (TT), and prothrombin time (PT) of patients with normal blood function, which typically range from 10 to 40 s, providing a physiologically relevant window for assessing the initiation of the coagulation dynamics.

We modeled either factor XI or factor XII anticoagulant treatment by inhibiting each of these factors’ initial concentration. We defined the inhibition level of factor i as

(10) $${\text{INH}}_{i}=\frac{u_{i}^{0}-u_{i}^{t}}{u_{i}^{0}},\;\text{for}\;i=\text{XI},\;\text{XII},$$

where $u_{i}^{0}$ is the nominal concentration (see Table 1) and $u_{i}^{t}$ is the inhibited target concentration. For the simulations presented in Section 3.4 we employed 9 inhibition levels of each factor: ${\text{INH}}_{i}=[0.25,0.50,0.70,0.75,0.80,0.85,0.90,0.95,0.975]$.

We employed two metrics to evaluate the initiation of the coagulation cascade in each patient-derived simulation. Given thrombin’s central role in coagulation, we defined the coagulation time $t_{co}$ as the moment thrombin concentration first exceeds a threshold concentration $u_{IIa}^{th}$ within the LAA. Additionally, we defined the coagulating volume as the volume in the LAA where this threshold is exceeded, namely

(11) $$V_{co}\left(t\right)=\int_{\Omega_{LAA}\left(t\right)}\phi \left(\vec{x},t\right)d\Omega_{LAA},$$

where $\Omega_{LAA}(t)$ represents all points $\vec{x}$ within the LAA, and $\phi (\vec{x},t)=1$ where $u_{IIa}(\vec{x},t)>u_{IIa}^{th}$, otherwise $\phi (\vec{x},t)=0$.

Following previous studies [41–43], we defined the threshold concentration as $u_{IIa}^{th}=2\;\text{nM}$, which roughly corresponds to the thrombin concentration at the transition from the initiation to the propagation phase. Thrombin concentration rises exponentially during the propagation phase (see Fig. 1F and 44), accelerating fibring formation and platelet activation. Consequently, any fluid volume surpassing $u_{IIa}^{th}$ poses a clotting risk. Larger coagulating volumes ($V_{co}$) indicate broader thrombin accumulation, which may promote fibrin deposition and sustained coagulation. In this work, we use the volume of these activated regions (i.e., the coagulating volume $V_{co}$), along with the coagulation time ($t_{co}$) and the maximum thrombin concentration over time as markers of elevated *prothrombotic risk* for patient-specific coagulation assessments.

### 2.3. CT imaging

We studied a group of N=13 subjects, selected to sample a wide range of anatomical and functional characteristics relevant to atrial fibrillation and thrombosis. Subjects 1–3 were enrolled at the National Institutes of Health (NIH) in Bethesda, Maryland (N = 3). Subjects 4–11 were enrolled at the University of California San Diego (UCSD), CA, United States (N = 8). Subjects 12 and 13 were enrolled at Hospital General Universitario Gregorio Marañón (HGUGM), Madrid, Spain.

Each of the 13 study subjects underwent 3D, time-resolved computed tomography scans (4D-CT, see Fig. 1A) to segment the LA anatomy. The voxel dimension ranged from 0.32 mm to 0.62 mm in the x–y plane and from 0.45 mm to 1 mm in the z direction. Time-resolved imaging data were obtained at regular intervals across the cardiac cycle, spanning from 5% to 10% of the R–R interval.

### 2.4. 4D personalized LA meshing

The LA computational meshes were generated in four steps using ITK-SNAP [45] and custom MATLAB scripts. Initially, the 3D LA anatomy was segmented from CT images, identifying key landmarks such as the pulmonary vein (PV) inlets, mitral annulus, and left atrial appendage (LAA). Then a triangular mesh was created for each LA segmentation [46], using the same spatial resolution selected for the CFD solver (see next section). These meshes were registered across the cardiac cycle to ensure coherence in vertex and centroid positions using the Coherent Point Drift algorithm [47]. The interpolation of the positions of the vertex and centroids to the time resolution of the CFD simulation was performed using a temporal Fourier series. Further details on image acquisition, reconstruction, and mesh generation can be found in [48].

### 2.5. Computational fluid dynamics

**Fig. 2.**
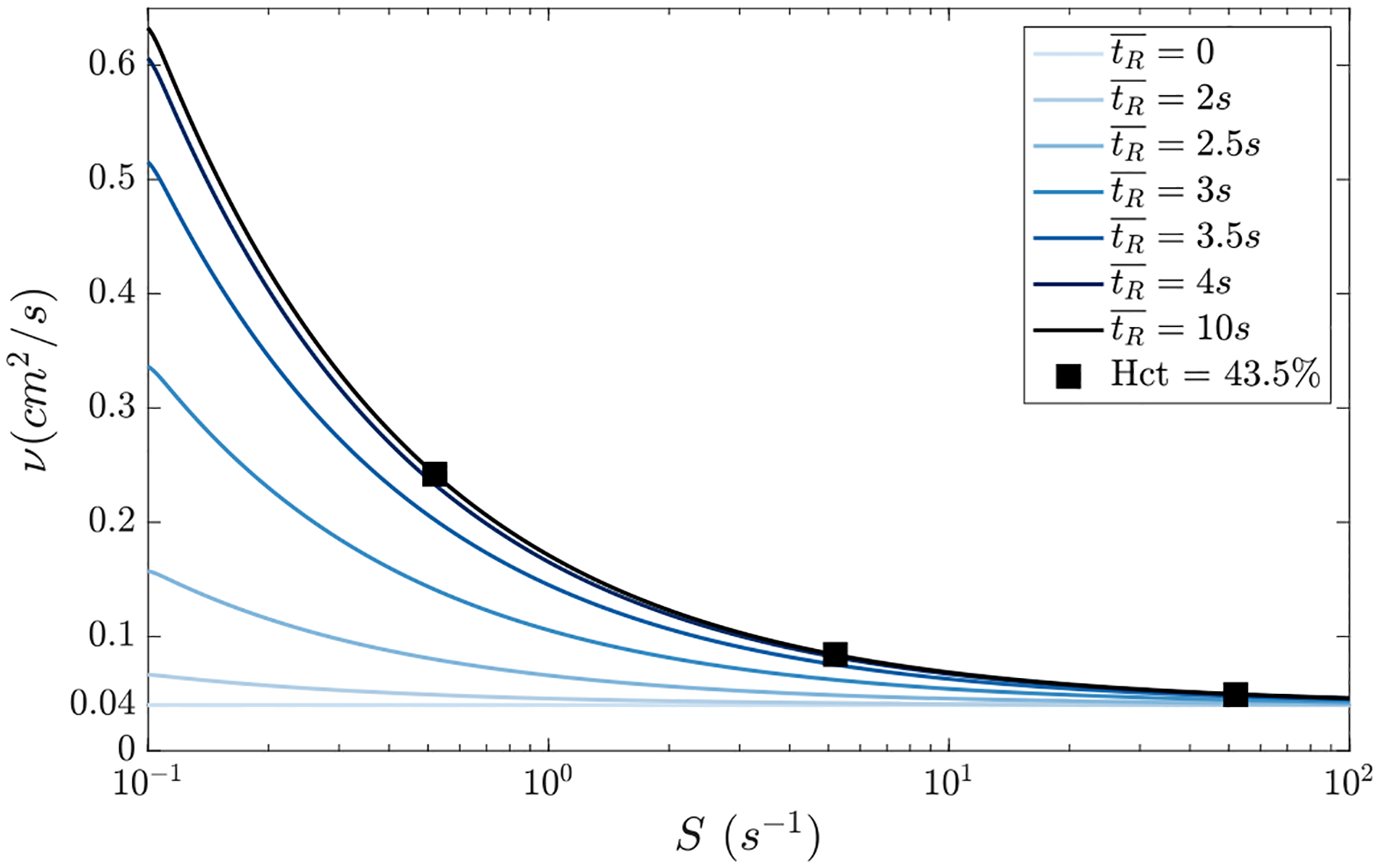
Residence-Time-Activated Carreau–Yasuda model: Non-Newtonian constitutive laws for the kinematic viscosity as a function of shear rate (S) and residence time ($\bar{t_{R}}$), denoted as $v(S,\bar{t_{R}})$ for the residence-time-activated Carreau–Yasuda model. The black squares represent data from [53] study for the respective hematocrit (Hct) value.

We adapted proprietary CFD code [49] to solve the Navier–Stokes equations for non-Newtonian incompressible flow

(12) $$\rho \frac{\partial \vec{v}}{\partial t}+\rho \vec{v}\cdot \nabla \vec{v}=-\nabla p+\nabla \cdot \bar{\bar{\tau }},$$

(13) $$\nabla \cdot \vec{v}=0,$$

where $\vec{v}$ and p are the velocity and pressure fields, ρ the fluid density, and $\bar{\bar{\tau }}$ the viscous stress tensor, using patient-specific LA meshes. We used a residence-time-activated Carreau–Yasuda model [50] to represent the thixotropic, shear-thinning rheology of blood arising from formation and rupture of RBC aggregates [51]. This model provides a non-Newtonian constitutive relation between blood viscosity v and shear rate S that depends on $\bar{t_{R}}$ as

(14) $$v\left(S,\bar{t_{R}}\right)=v_{\infty }+H\left(\bar{t_{R}}\right)\left(v_{0}-v_{\infty }\right){\left(1+(\lambda S{)}^{a}\right)}^{\frac{n-1}{a}},$$

(15) $$H\left(\bar{t_{R}}\right)=\frac{\left.1+\text{erf}\;\left[\bar{\left(t_{R}\right.}-t_{\mu }\right)/\sqrt{2}\sigma \right]}{2},\;t_{\mu }=3s,\;\sigma =0.6s,$$

where $t_{\mu }$ are timescales associated to RBC aggregation, and $\lambda =8.2,a=24.32,n=0.37,v_{0}=16v_{\infty }$ and $v_{\infty }=0.04{\;\text{cm}}^{2}/\text{s}$ are the Carreau–Yasuda model constants. These constants depend on the hematocrit (*Hct*), and the values chosen for our simulations correspond to Hct=43.5, which falls within the physiological range [52]. Fig. 2 illustrates the impact of the residence time and shear on the viscosity, showing that non-Newtonian effects are negligible for $\bar{t_{R}}≲2\;\text{s}$, gradually increasing for $2\;\text{s}≲\bar{t_{R}}≲10\;\text{s}$. For $\bar{t_{R}}≳10\;\text{s}$ the kinematic viscosity provided by the residence-time-activated Carreau–Yasuda model becomes indistinguishable from its classic version.

Each patient-derived LA simulation was run for 20 cardiac cycles with a fixed time step Δt, chosen to ensure a Courant–Friedrichs–Lewy (CFL) number below 0.3 throughout the complete run. The fluid domain was discretized using a staggered Cartesian grid with a uniform spacing of Δx=0.051mm. As previously reported in [48], this resolution has been shown to accurately capture atrial hemodynamics, a conclusion further supported by Khalili et al. [54]. The spatial derivatives were approximated using second-order centered finite differences. The segmented LA geometry was embedded within a 13-cm cubic domain with periodic boundary conditions. The LA surface motion, derived from patient-specific 4D CT images, was prescribed throughout the cardiac cycle and influenced flow via the no-slip boundary condition, which was enforced using the immersed boundary method (IBM) proposed by Uhlmann [55].

Inflow boundary conditions were imposed assuming equal flow rate through each pulmonary vein (PV), denoted as $Q_{PV,i}$, for i=1…4 (see Fig. 1B). Specifically,

(16) $$Q_{PV,i}\left(t\right)=\frac{1}{4}\left(\frac{dV_{LA}}{dt}-Q_{MV}\left(t\right)\right)\;\text{and}\;Q_{MV}\left(t\right)=\text{max}\left(\frac{dV_{LV}}{dt},0\right),$$

where $V_{LA}$ represents the time-dependent volume of the LA, $Q_{MV}$ denotes the flow rate through the mitral valve, and $V_{LV}$ is the left ventricle (LV) volume obtained from the CT image. To enforce the velocity $v_{i}$ at each PV, a cylindrical buffer region was added upstream of each PV inlet plane. A volumetric force was added in this buffer region, using a variation of the formulation of the IBM model. Further details can be found in [48].

Boundary conditions at the mitral valve outlet were applied to the plane section at the downstream end of the atrial segmentation, which dynamically moved within the cubic simulation domain as the LA walls deformed. When the mitral valve was closed, mesh points in that section were treated as a standard no-slip boundary, identical to the rest of the atrial wall. Conversely, when the mitral valve was open (i.e., $Q_{MV}>0$), no boundary condition (i.e., no IBM forcing) was imposed on these mesh points.

Our CFD code solved simultaneously the Navier–Stokes equations to produce velocity and pressure fields (Fig. 1C), and the transport Eqs. (3)–(5) for the residence time and higher order moments (Fig. 1D). To address the lack of diffusion on Eqs. (3), (4), and (5), a third-order weighted essentially non-oscillatory (WENO) scheme [56] was used to compute the non-linear terms [48,50,57]. This scheme prevents spurious oscillations in the numerical solutions while minimizing the overall numerical diffusivity. For the MuFi approach, the concentration field $u_{i}(\vec{x},t)$ was mapped from the residence time and higher order moments using Eqs. (6), (7) or (8), with the values of $g_{i},g_{i}^{\prime }$ and $g_{i}^{\prime \prime }$ obtained from the no-flow ODE systems described in Section 2.2.

### 2.6. Verification of MuFi modeling in 3D patient-specific anatomies

We solved the HiFi advection–diffusion–reaction Eqs. (2) in 3D patient-specific LA anatomies and compared the results of the MuFi and HiFi models. Given the high cost of running the HiFi system, we made several arrangements to reduce compute time. Similar to our previous work [23], we used the 9-species system of Zarnitsina et al. [58] instead of the 32-species of Zhu [24] with the reaction rates and initial conditions described in Appendix A.2. Instead of performing the verification analysis for all patients, we selected two subjects representative of normal and impaired atrial function.

We discretized the HiFi system of PDEs similar to the PDEs governing the residence time, including a WENO scheme for the non-linear terms. However, to further reduce computational time, the HiFi system was not integrated together with the flow in the CFD solver. Instead we solved the incompressible Navier–Stokes equations (Eq. (12)) over 10 cycles to ensure a quasi-time-periodic flow. Then, we phase averaged the last 5 cycles, and stored 40 3D velocity fields, i.e., one field every 500 time steps. Subsequently, we solved the HiFi PDE system interpolating the phase-averaged velocity linearly in time. To ensure an unbiased comparison between the HiFi and MuFi models, the residence time and higher-order moments used in the verification study were obtained solving transport Eqs. (3), (4) and (5) with linearly interpolated velocity fields in time. For all other results reported in this manuscript, we used the full-resolution residence time fields integrated concurrently with the flow in the CFD solver, as described in Section 2.5 above.

The global relative error of the MuFi models inside the LAA was quantified as

(17) $$\varepsilon_{IIa}^{\text{max}}(t)=\underset{\vec{x}\in \Omega_{\text{LAA}}}{\text{max}}\left(\frac{\left|u_{IIa}^{\text{MuFi}-p}(\vec{x},t)-u_{IIa}^{\text{HiFi}}(\vec{x},t)\right|}{u_{IIa}^{\text{HiFi}}(\vec{x},t)}\right),$$

where $\Omega_{\text{LAA}}$ represents the volume of the LAA at each time step of the simulation. We focused on the LAA because this is the site of maximum thrombin concentration and most likely site of atrial thrombosis.

## 3. Results

### 3.1. Patient characteristics

**Fig. 3.**
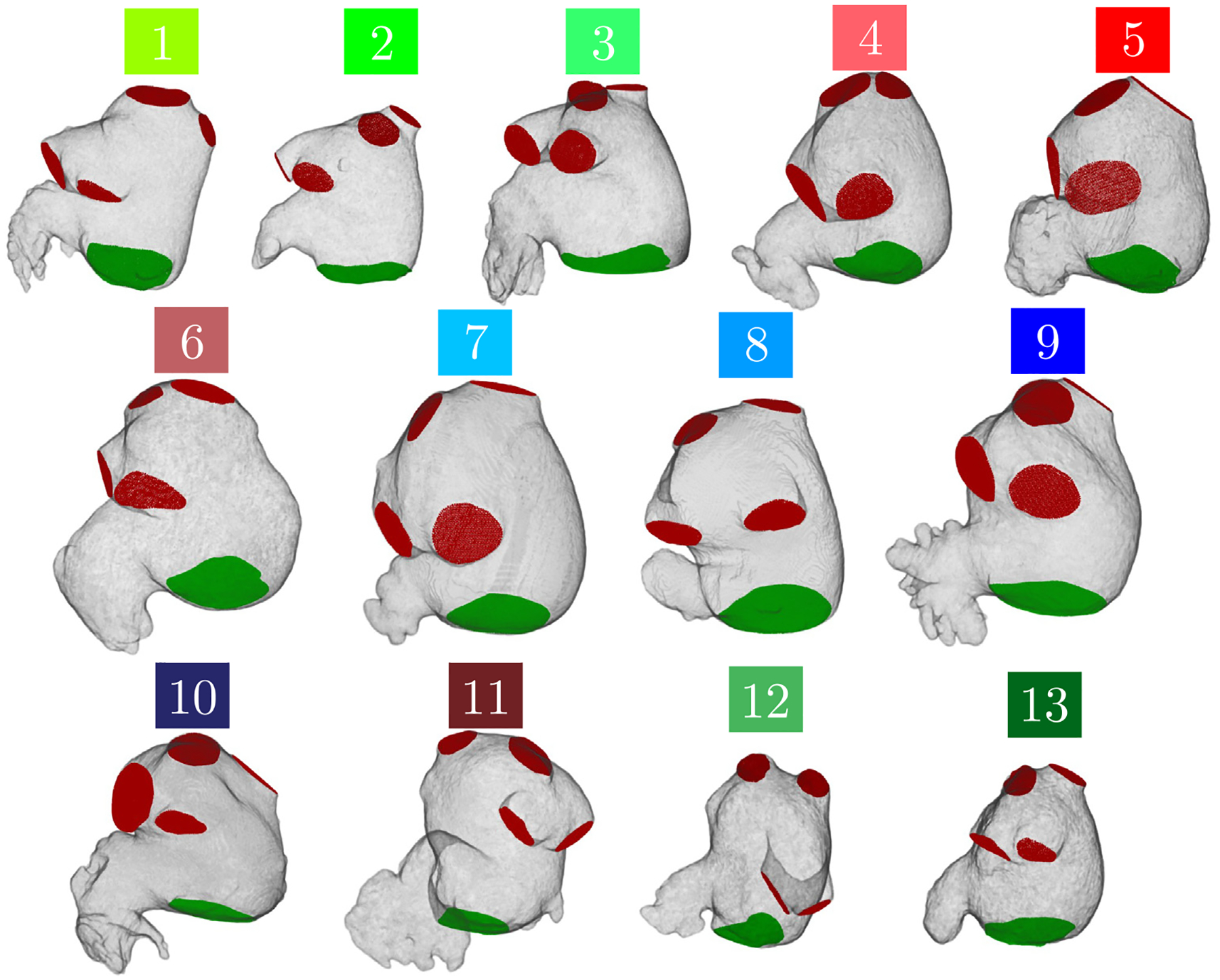
Patient-Specific Left Atrium Meshes: Three-dimensional Lagrangian mesh derived from Computerized Tomography (CT) scans depicting the left atrium walls and pulmonary veins (PVs highlighted in red) as well as the mitral valve outlet surfaces (in green). These images represent a moment at the start of the R–R interval.

Table 2 summarizes the baseline demographic characteristics of the study cohort. We included 13 subjects (median [25–75 percentile] age 65 [57–82] years, 7 [54%] males). Seven (54%) subjects (4–11) had AF, 6 of them had their CT obtained in AF while one subject (10) was in sinus rhythm. Additionally, 3 subjects (5, 6, and 11) had an LAA thrombus, which was digitally removed from the segmentations before running the CFD simulations, and one subject (4) did had a history of transient ischemic attack (TIA). Based on these data, we separated the subjects in 3 groups: 1) those without prior AF nor thromboembolic events (1–3, 12–13); 2) those with AF but without thromboembolic events (7–10) and 3) those with thromboembolic events (4–6 and 11). The average normalized residence time in the LAA of each patient, ${\left\langle \bar{t_{R}}\right\rangle }_{\text{LAA}}/t_{c}$, was lowest in group 1 (mean ± STD = 2.09 ± 0.33), while groups 2 and 3 had comparable values (5.13 ± 2.11 and 4.59 ± 0.96, respectively). Fig. 3 shows the segmented LA anatomies of the 13 subjects at the onset of the R–R interval, including inlet (PVs) and outlet (mitral annulus) sections. The color assigned to each subject is based on the grouping described above: green for group 1, blue for group 2 and red for group 3.

### 3.2. Comparison of multi-fidelity and high-fidelity coagulation models

Multi-fidelity (MuFi) modeling of advection–diffusion–reaction processes in the low-diffusivity limit has shown promise as a means to significantly accelerate the simulation of blood coagulation under flow [23]. However, this approach has not been verified yet in realistic 3D cardiovascular geometries. This section compares HiFi and MuFi simulations for 3D patient-derived LA flows. Due to the high computational cost of the HiFi simulations with the 32-species model described in Section 2.2, we chose a smaller 9-species model for the MuFi verification. This choice is justified by our previous work [23], which shows that the main source of uncertainty in the MuFi model arises from the maximum growth rates, and not from the number of species involved in the coagulation cascade model.

Two representative patients were selected for the verification of the MuFi model: case 2 from the LAA-thrombus-negative group and case 6 from the LAA-thrombus-positive group. The initial and inlet conditions for the concentrations of the 9 species are provided in Table 3. The initial thrombin concentration is set at an artificially high level to ensure that peak thrombin concentration ($u_{IIa}\approx 700\;\text{nM}$) in this 9-species model is achieved within 20 cardiac cycles. Hence, this analysis is not intended for thrombus risk evaluation, but rather to provide a controlled benchmark for validating the computational approach.

**Fig. 4.**
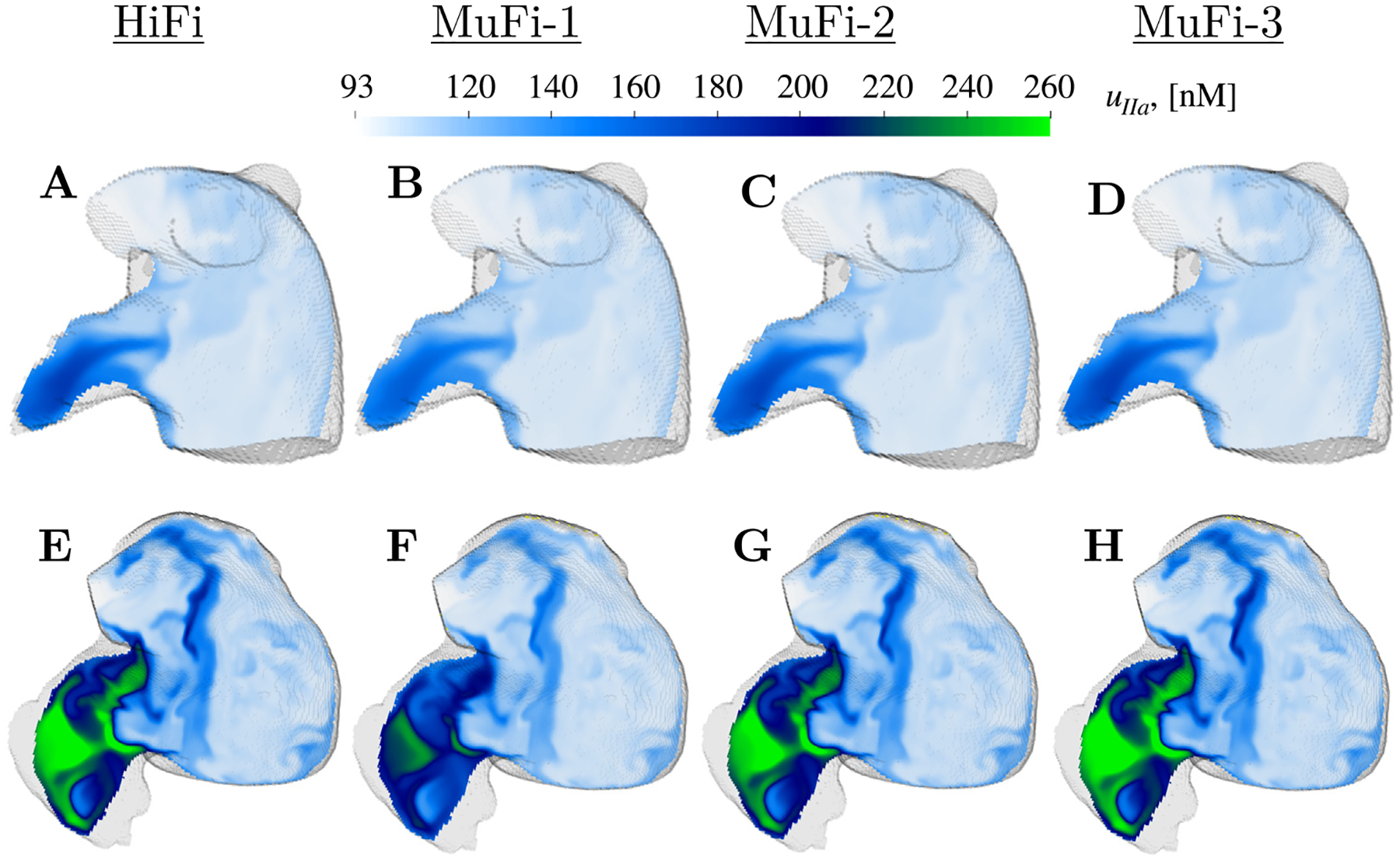
Spatial distribution of thrombin concentration $u_{IIa}$ on oblique plane sections: Case 2 (top) and case 6 (bottom) at $t/t_{c}=20$. HiFi (A,E), Mufi-1 (B,F), MuFi-2 (C,G), MuFi-3 (D,H).

Fig. 4 depicts the spatial distributions of the thrombin concentration, $u_{IIa}$, for the HiFi model and the MuFi-1, MuFi-2, and MuFi-3 models. The data are represented at early atrial diastole of the 20^th^ simulation cycle. Consonant with the HiFi model, the three MuFi models resolved the thrombin concentration peaks in the LAA and predicted significantly higher $u_{IIa}$ in the thrombus-positive subject (Fig. 4A–D) than in the thrombus-negative one (Fig. 4E–H). Nevertheless, MuFi-1 tended to underestimate the peak values of $u_{IIa}$, whereas MuFi-2 and MuFi-3 produced $u_{IIa}$ distributions in closer agreement with the HiFi results.

**Fig. 5.**
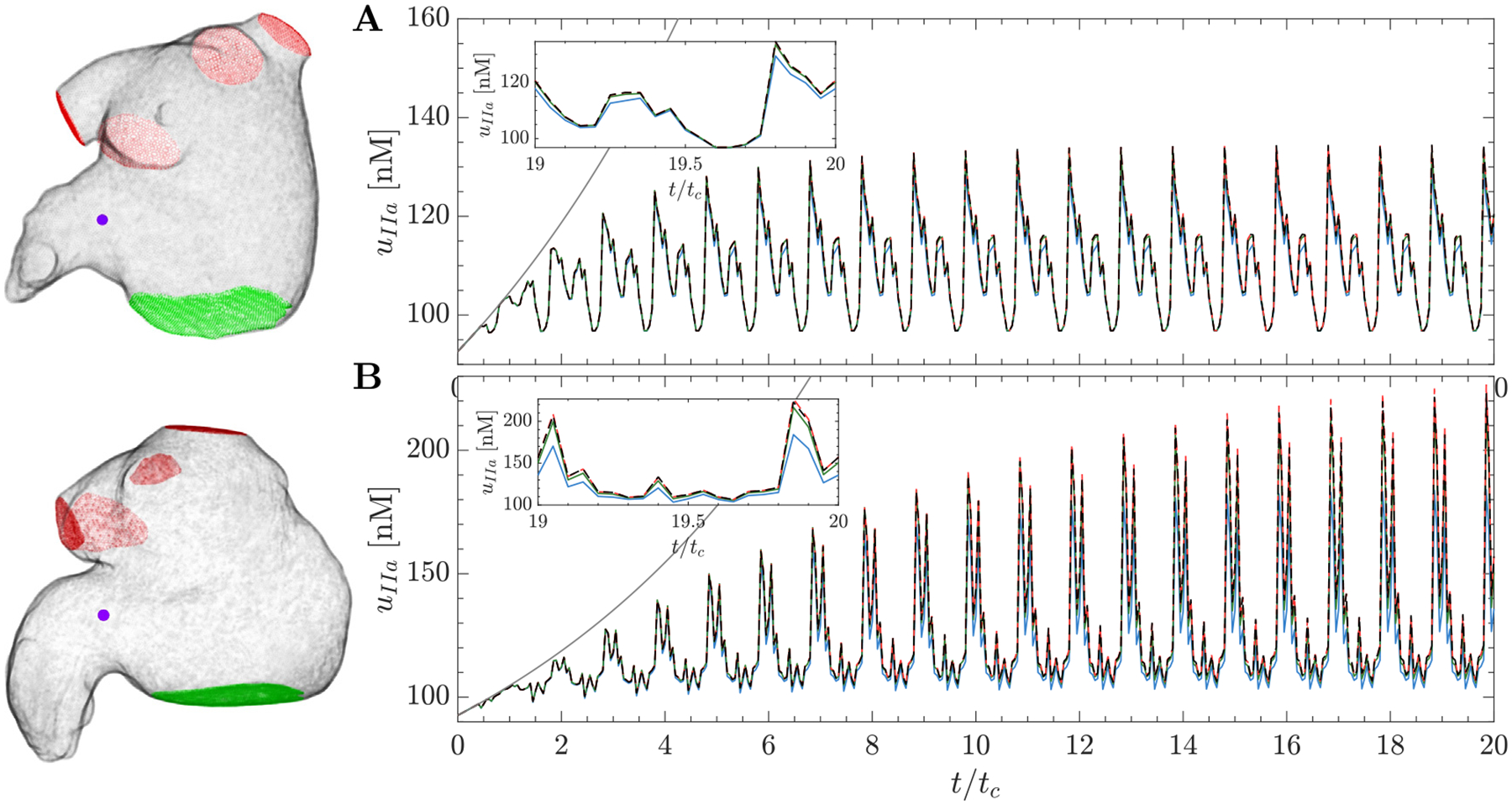
Thrombin concentration, $u_{II_{a}}$, versus normalized time, $t/t_{c}$: Case 2 (A) and Case 6 (B). Each line corresponds to a different model: HiFi (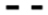), MuFi-1 (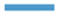), MuFi-2 (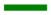) and MuFi-3 (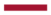). The locations are considered in the near region of the ostium plane and indicated with (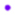). For reference, the solution of the 9-species no-flow reaction model (Eq. (9)) is also included (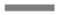). The inset in each panel shows the evolution of $u_{IIa}$ during the 20th cycle.

To evaluate the MuFi models in more detail, we also compared the temporal evolutions of $u_{IIa}$ predicted at the ostium center for subjects 2 and 6 (Fig. 5). In these plots, the 9-component no-flow reaction model was also included for reference (solid line). The HiFi and all MuFi models departed from the no-flow model quickly after one cardiac cycle, suggesting that no-flow models severely overestimate thrombin concentrations for timescales relevant to the coagulation process. Thrombin concentration in the HiFi and MuFi models experienced oscillations with a slowly growing envelope due to the cyclic inflow of “fresh” low-$u_{IIa}$ blood. In subject 2, who had sinus rhythm and normal LA function, this fluid exchange was more vigorous and, consequently, the intra-cycle peak value of $u_{IIa}$ stopped growing at $t\approx 8t_{c}$ (Fig. 5A). On the other hand, the envelope of $u_{IIa}$ was still growing slowly at $t=20t_{c}$ for subject 6, who had AF and low LA function (Fig. 5B). Overall, all the MuFi models captured the temporal dynamics of $u_{IIa}$ regardless of their order of approximation. Consistent with the results in Fig. 4, MuFi-1 tended to underestimate the peak values of $u_{IIa}$ after integrating the models for 20 cardiac cycles. These differences were more significant in the patient exhibiting higher thrombin concentrations. The MuFi-2 model showed good agreement with the HiFi solution for both subjects. The MuFi-3 followed the HiFi results slightly better than MuFi-2.

**Fig. 6.**
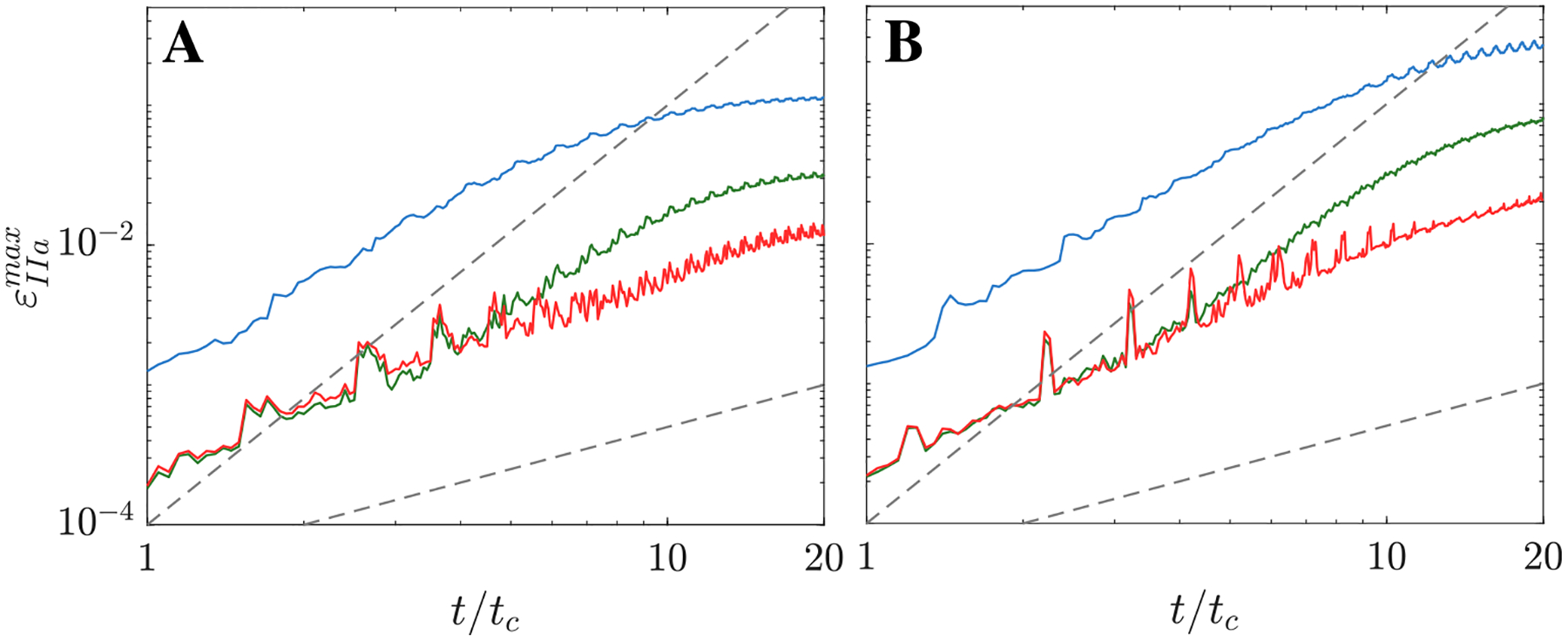
MuFi Maximum relative error in the LAA, $\varepsilon_{IIa}^{max}$, vs. normalized time, $t/t_{c}$ : Case 2 (A) and Case 6 (B). Each line corresponds to a different model: MuFi-1 (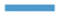), MuFi-2 (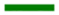) and MuFi-3 (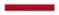). Dashed lines correspond to $\varepsilon_{IIa}^{\text{max}}\propto \left(t/t_{c}\right)$ and $\varepsilon_{IIa}^{\text{max}}\propto {\left(t/t_{c}\right)}^{3}$.

Fig. 6 displays the time evolution of the global relative error of the MuFi models, $\varepsilon_{IIa}^{\text{max}}$ defined in Eq. (17), for the three MuFi models and two subjects discussed above. In both cases, MuFi-1 exhibited markedly larger errors than MuFi-2 and MuFi-3 at all times, reaching $\varepsilon^{max}\sim 10\%$ in case 2 and 25% in case 6 by the 20th cycle. The MuFi-2 and MuFi-3 errors differed less from each other and were smaller than 0.3% for the first 5 cardiac cycles. For $t≳5t_{c}$, the MuFi-2 error grew faster with time, reaching $\varepsilon^{max}\sim 3\%$ and 8% for subjects 2 and 6 at $t=20t_{c}$, while the MuFi-3 error was $\varepsilon^{\text{max}}\sim 1\%$ and 2% for the same subjects.

In terms of computational cost, solving 20 cardiac cycles of the 9-equation coagulation system using the HiFi model took approximately 320 min using the Python version of the solver with Numba CUDA, as detailed in [59], on a GPU A100 with 80 GB of RAM, 6912 CUDA cores, and 432 tensor cores. In comparison, on the same hardware, the MuFi-1 approach took approximately 35 min, while the MuFi-2 and MuFi-3 models ran in 45 min and 72 min. Based on these data, the MuFi-2 model was deemed to provide the best balance between accuracy and computational cost, and all the subsequent analyses presented in this manuscript were performed with this model.

### 3.3. Patient-derived models of LA coagulation cascade initiation

This section examines the progression of thrombin concentration in all the 3D patient-derived LA models by applying a MuFi-2 model with 32-components kinetics. The analysis used the nominal initial concentrations outlined in Table 1 and temporal integration was performed over 20 cardiac cycles.

**Fig. 7.**
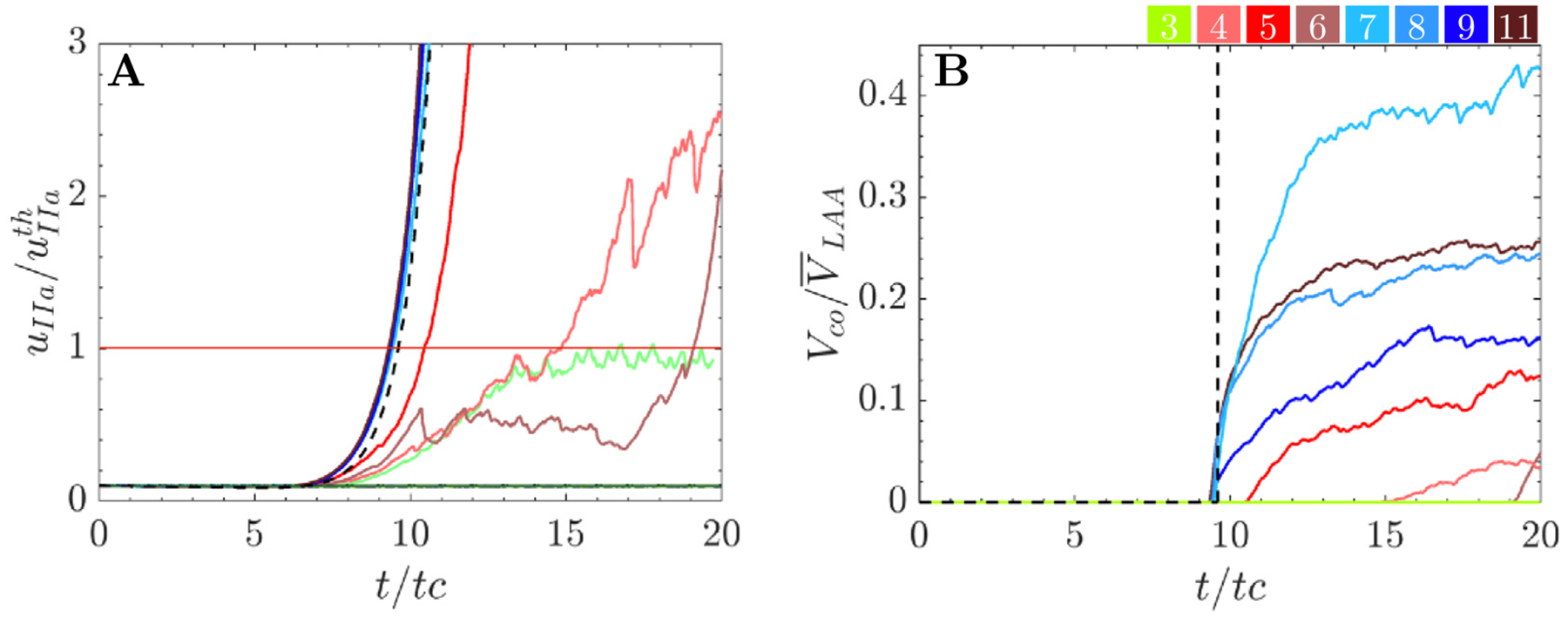
Temporal evolution of thrombin concentration and coagulating volume in the LAA: (A) Normalized maximum thrombin concentration ($u_{IIa}/u_{IIa}^{\text{th}}$) in each subject’s the LAA versus normalized time ($t/t_{c}$). The no-flow solution (Eq. (9)) is provided for reference (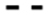). (B) Coagulating volume within the LAA ($V_{co}$) normalized by mean LAA volume. Line colors are defined in Fig. 3.

Fig. 7A illustrates the temporal evolution of the maximum thrombin concentration within the LAA normalized with the threshold concentration value, $u_{IIa}^{th}=2\text{nM}$. Fig. 7B displays the temporal evolution of the LAA coagulating volume, $V_{co}$ normalized by the mean LAA volume, $V_{LAA}$. As before, the results from the no-flow ODE model were included for reference in Fig. 7A–B (dashed line). In this model, the thrombin concentration started to grow exponentially in the 8th cardiac cycle, reaching the threshold concentration at $t\approx 9.6t_{c}$ (Fig. 7A). Consequently, $V_{co}/V_{LAA}$ adopted the shape of a step function that jumped from zero to one at that time point (Fig. 7B).

By comparing each subject’s LAA thrombin dynamics with the no-flow results, we identified three distinct coagulation behaviors (Fig. 7A–B). A first group of subjects that we defined as having *no prothrombic* risk in the atrium (cases 1, 2, 10, 12, and 13), exhibited virtually no activation of the coagulation cascade, with nearly constant thrombin values around the initial concentration (horizontal lines in Fig. 7A). As a result, all these cases had zero $V_{co}/V_{LAA}$ over the course of the simulations (Fig. 7B). A second group was considered to have *moderate prothrombotic risk* in the atrium (cases 3, 4 and 6). These subjects had more or less intricate patterns of sub-exponential growth and fluctuations in $u_{IIa}$, and non-zero albeit small values of $V_{co}/V_{LAA}$ by $t=20t_{c}$ (Fig. 7B). The third group of subjects were considered to have *high prothrombotic risk* in the atrium (cases 5, 7, 8, 9 and 11). They experienced nearly exponential growth with in thrombin concentration similar to the no-flow model (Fig. 7A). Accordingly, the evolution of their $V_{co}/V_{LAA}$ over time resembled a step function despite some small intra-cycle fluctuations (Fig. 7B). The normalized LAA residence times of the *no-risk, moderate-risk* and *high-risk* groups were in average 2.12 ± 0.33, 3.61 ± 1.45 and 5.58 ± 1.26 cycles, respectively. Importantly, the prothrombotic risk classification derived from the maximum thrombin concentration was rather insensitive to the thrombin threshold. For instance, the group assigned to all but one patients would remain unchanged when varying this threshold between 1 and 4 nM. The only exception, subject 3, would switch from no *risk* to *moderate risk*.

**Fig. 8.**
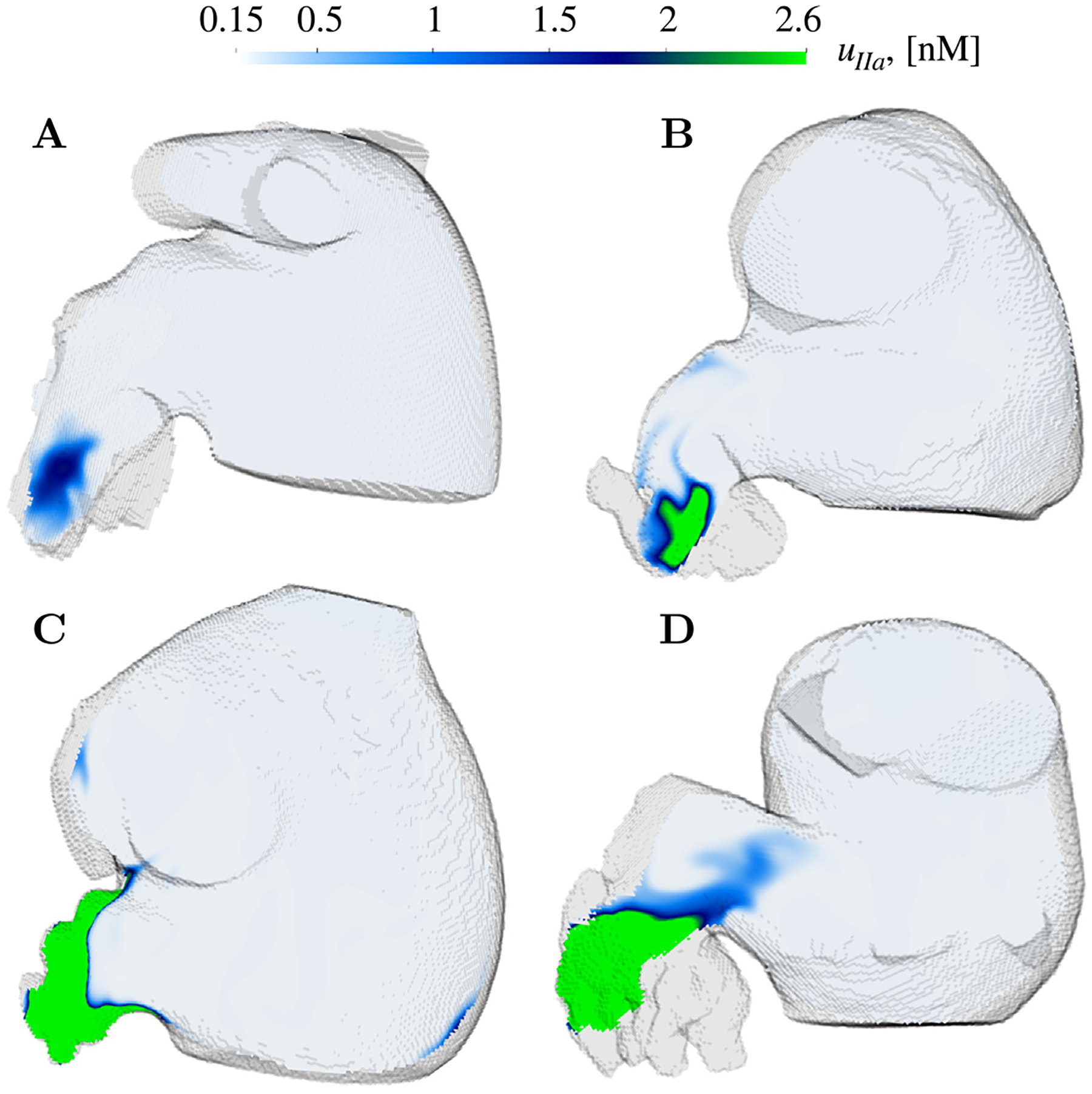
Spatial distribution of thrombin accumulation ($u_{IIa}$) in oblique plane sections: Thrombin concentration fields ($u_{\text{IIa}}$) in oblique plane sections intersecting the LAA for *moderately coagulating* cases 3 (A) and 4 (B), and *severely coagulating* cases 7 (C) and 11 (D), after 20 cardiac cycles $t/t_{c}=20$. The color scheme has a sharp jump between dark blue and bright green at $u_{IIa}=2\;\text{nM}$ to facilitate visualizing each patient’s $V_{co}$.

Fig. 8 displays the LAA thrombin distribution at $t=20t_{c}$ in representative cases of the moderate and *high prothrombotic risk* groups. We used a color scheme with a sharp gradient at the thrombin threshold $u_{IIa}^{th}=2$ nM to help visualize each patient’s $V_{co}$. We did not include thrombin maps for no-risk subjects. These maps were not informative as $u_{IIa}$ remained significantly lower than $u_{IIa}^{\text{th}}$ throughout the LA. A noticeable yet moderately sized region of elevated thrombin concentration was observed in the distal LAA of the *moderate prothrombotic risk* cases (Fig. 8A and B). In contrast, two of the cases with a *high prothrombotic risk* had voluminous areas of above-the-threshold thrombin concentration that occupied significant portions of the LAA (Fig. 8C and D).

### 3.4. Patient-derived models of factor XI/XII inhibition of the coagulation cascade

We leveraged the computational efficiency of MuFi models to systematically investigate how factor XI/XII inhibition affects LA coagulation under patient-specific flow patterns. Inhibition was modeled by lowering these factors’ initial concentrations from the nominal values shown in Table 1. For each subject, we examined no inhibition and 9 different inhibition levels applied to non-active factors XI or XII, as defined by varying the parameter ${\text{INH}}_{i}$ (Eq. (10) in §2.2) in the range [0.25, 0.975].

**Fig. 9.**
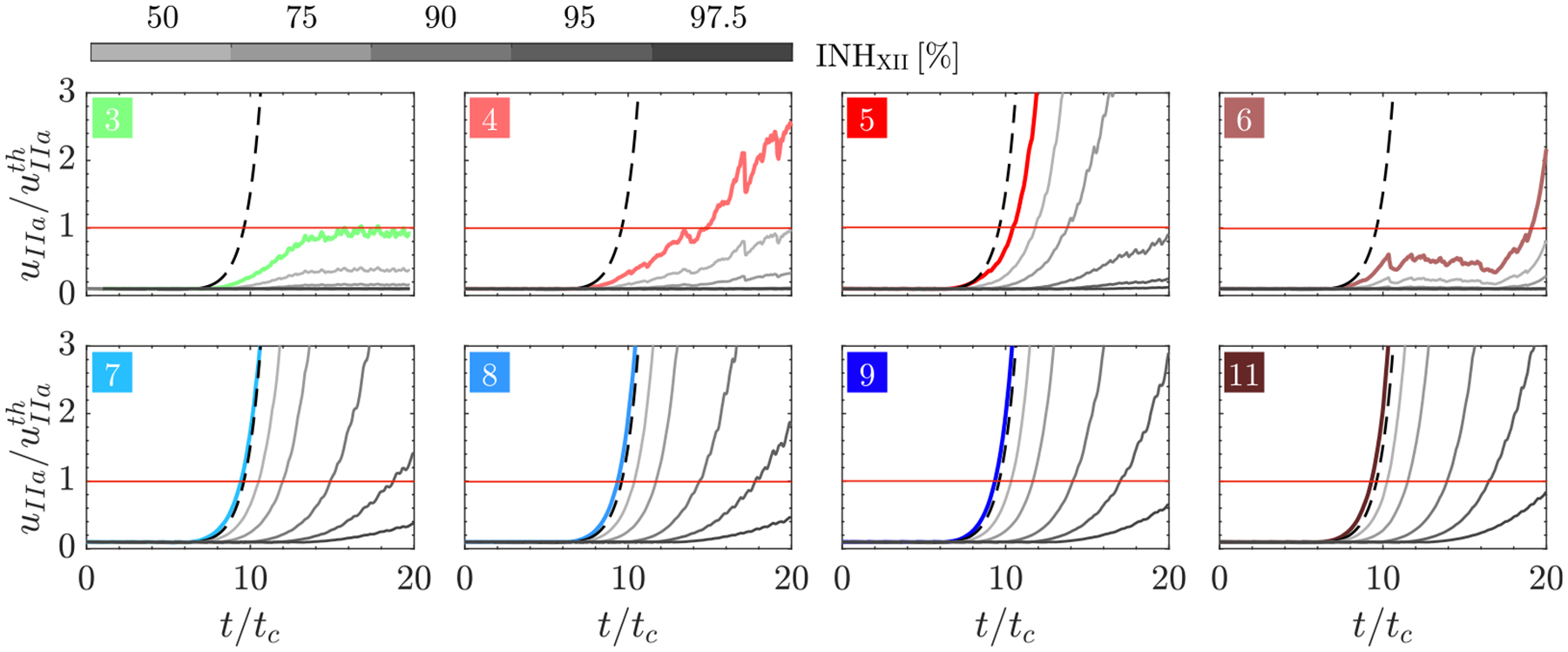
Temporal evolution of maximum thrombin concentration ($u_{IIa}$) in the LAA across Factor XI Inhibition Level: Time series depicting the maximum thrombin concentration $\left(u_{IIa}\right)$ for the nominal case (color lines) and five inhibition levels for factor XI: ${\text{INH}}_{XI}=[50,75,90,95,97.5]\%$ (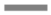) in the LAA of each patient, normalized by the thrombin concentration threshold ($u_{IIa}^{th}=2\;\text{nM}$). Additionally, the no-flow solution of the 32-ODE system Eq. (9) is provided for reference (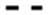).

**Fig. 10.**
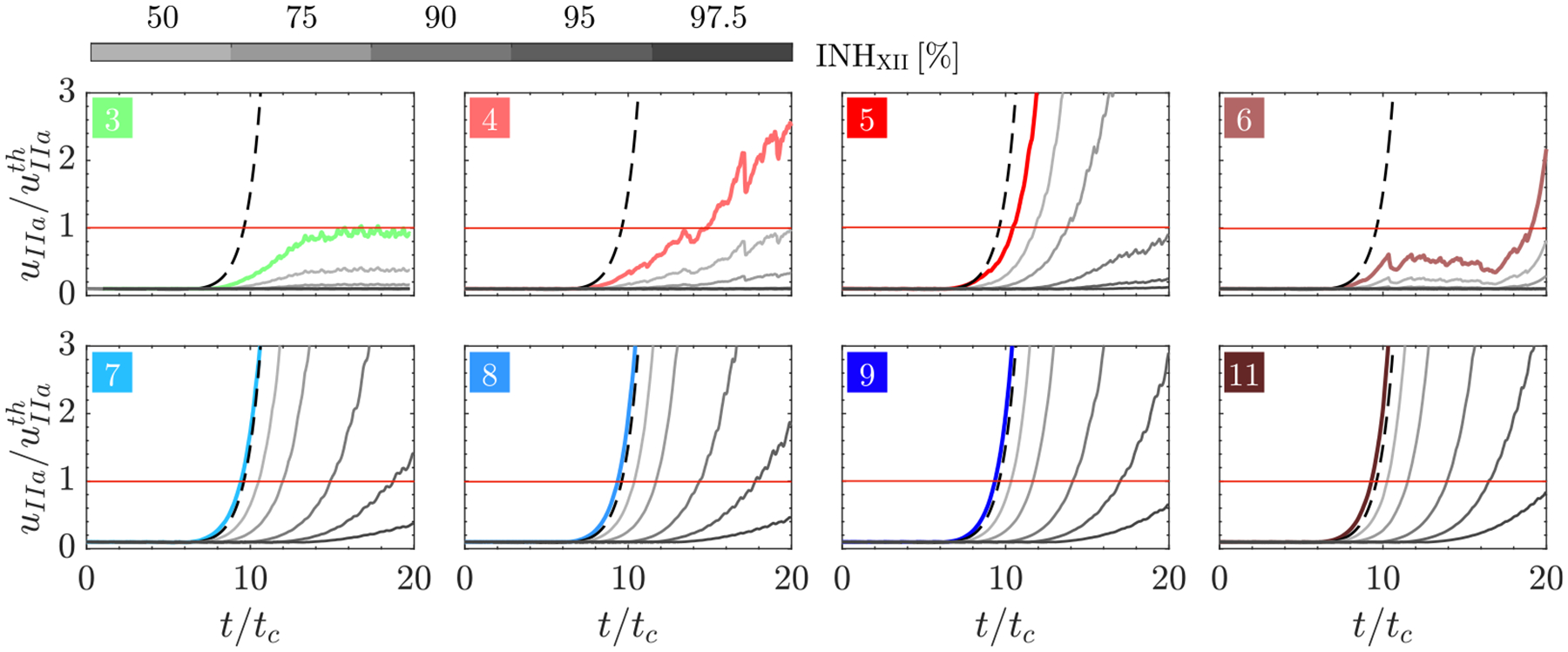
Temporal evolution of maximum thrombin concentration ($u_{IIa}$) in the LAA across Factor XII Inhibition Level: Time series depicting the maximum thrombin concentration ($u_{IIa}$) for the nominal case (color lines) and five inhibition levels for factor XII: ${\text{INH}}_{XII}=[50,75,90,95,97.5]\%$ (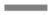) in the LAA of each patient, normalized by the thrombin concentration threshold ($u_{IIa}^{th}=2\;\text{nM}$). Additionally, the no-flow solution of the 32-ODE system Eq. (9) is provided for reference (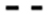).

Figs. 9 and 10 show the time evolution of the maximum thrombin concentration within the LAA for varying inhibition levels of factors XI and XII. In these figures, we only included data from those patients exhibiting appreciable thrombin concentration after 20 cycles of simulation time, i.e., the *moderate* and *high prothrombotic risk* groups described above. For reference, the plots include the results from the flow (colored lines) and no-flow (black dashed lines) models without inhibition. In all cases shown, the maximum thrombin concentration was normalized by the threshold value ($u_{IIa}^{th}=2\text{nM}$). Interestingly, factor XI/XII inhibition had varying effects on LAA thrombin dynamics for different subjects. For instance, in the *moderate prothrombotic risk* group (cases 3, 4, and 6), the raise of thrombin concentration was markedly blunted by mild inhibition $\left({\text{INH}}_{i}\leq 0.5\right)$ of either coagulation factor. On the other hand, the *high prothrombotic risk* group was less sensitive to inhibiting factor XI/XII. The patients in this group exceeded the threshold $u_{IIa}^{th}$ within the 20 simulated cycles for all factor XI inhibition and most factor XII inhibition levels considered.

When comparing factor XI and factor XII, we found that factor XII inhibition was more effective, particularly in the *high prothrombotic risk* group. Of note, Fig. 10 suggests that the coagulation threshold was not reached after the 20 cycles of simulation under sufficiently strong factor XII inhibition, even in this *high prothrombotic risk* group.

**Fig. 11.**
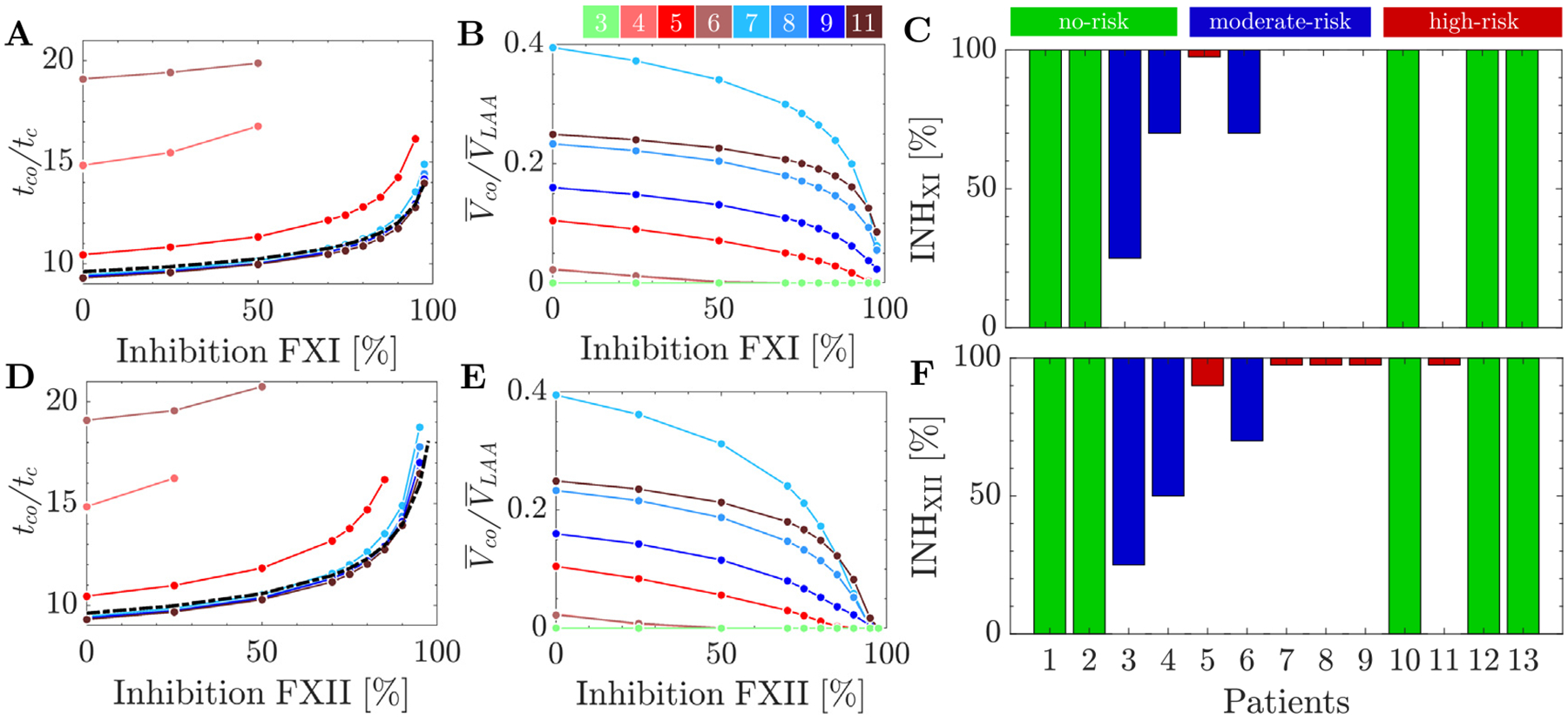
Effect of factor XI and factor XII inhibition levels on coagulation time and volume: (A, B) Coagulation time and mean coagulating volume in the last 5 cardiac cycles (normalized by the mean LAA volume) vs. factor XI inhibition level. (C) Factor XI inhibition range necessary to keep $u_{IIa}<u_{IIA}^{\text{th}}$. (D, E) Coagulation time and mean coagulating volume in the last 5 cardiac cycles (normalized by the mean LAA volume) vs. factor XII inhibition level. (F) Factor XII inhibition range necessary to keep $u_{IIa}<u_{IIA}^{th}$. The coagulation time obtained in the no-flow reaction model (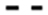) is shown as a reference in panels A and D.

To obtain summary metrics of the impact of factor XI/XII inhibition on coagulation in each subject, we plotted coagulation time ($t_{co}$) and coagulating volume ($V_{co}$) vs. each factor’s inhibition level (Fig. 11). In these plots, $V_{co}$ was averaged over the last simulation cycles and normalized by each subject’s mean LAA volume ($V_{LAA}$). Fig. 11A–B illustrate that after >50% inhibition of factor XI, cases 4 and 6 (with *moderate prothrombotic risk*) did not activate the coagulation cascade within the simulated 20 cycles (i.e., $t_{co}>20t_{c}$ and $V_{co}=0$). In contrast, all cases in the *high prothrombotic risk* group (5, 7, 8, 9, and 11) exhibited coagulation times consistent with the no-flow model. These times were relatively insensitive to factor XI inhibition levels ≲ 90% (Fig. 11A) and $V_{\text{co}}$ only decreased significantly when factor XI inhibition exceeded ≳ 90%. The coagulation time and volume displayed similar trends with factor XII inhibition (11D–E). However, factor XII inhibition prolonged $t_{co}$ and reduced $V_{co}$ more effectively than factor XI inhibition. In particular, subjects on the *high prothrombotic risk* group experienced more dramatic drops in coagulating volume for moderate values of factor XII inhibition (${\text{INH}}_{XII}\approx 75\%$). The response to anticoagulation did not correspond completely with the risk of coagulation under baseline conditions, as reflected by some of the $V_{co}$ vs. INH curves crossing each other. For example, the patient with largest normalized $V_{co}$ in the cohort at baseline (case 7) did not have the largest normalized $V_{co}$ under maximum inhibition of factor XI or factor XII (Fig. 11B,E).

Fig. 11C,F display the required values of factor XI/XII inhibition to bring thrombin concentration below the threshold level. These plots illustrate that 1) cases of the *no prothrombotic risk* group did not need inhibition, 2) coagulation can be prevented with moderate levels of factor XI/XII inhibition in patients with *moderate prothrombotic risk*, and 3) a full (for factor XI) or almost full (for factor XII) inhibition is required to prevent thrombus formation in the LAA in the *high prothrombotic risk* cases.

**Fig. 12.**
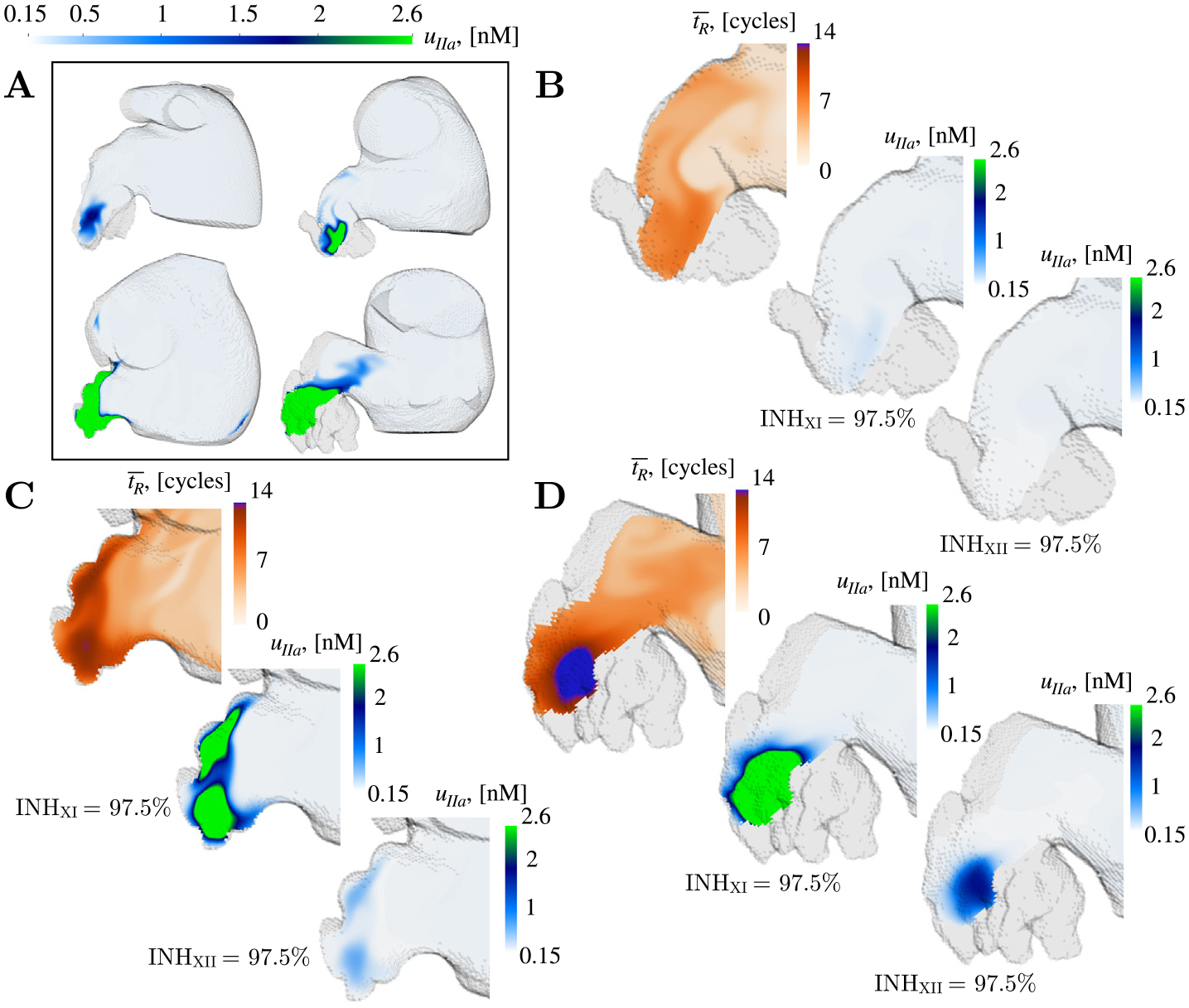
Spatial distribution of residence time $\left(\bar{t_{R}}\right)$ and thrombin accumulation $\left(u_{IIa}\right)$ on oblique plane sections after factor XI and XII inhibition: For reference, (A) displays $u_{IIa}$ for nominal initiation in cases 3,4,7 and 11. Spatial visualization of residence time ($\bar{t_{R}}$) and thrombin accumulation ($u_{IIa}$) for ${\text{INH}}_{XI}={\text{INH}}_{XII}=97.5\%$ on oblique plane sections for Case 4 (B), Case 7 (C) and Case 11 (D) after 20 cardiac cycles $t/t_{c}=20$.

Finally, we investigated whether the differential response to anticoagulation observed across different patients was related to patient-specific LAA blood flow patterns and stasis. Fig. 12 depicts the spatial distribution of residence time and thrombin inside the LAA of a subject with *moderate prothrombotic risk* (panel B) and two subjects with *high prothrombotic risk* (panels C and D) after 97.5% inhibition of factors XI/XII. In regions where $\bar{t_{R}}<10t_{c}$, both treatments successfully deactivated the coagulation cascade. Blood pools with quasi-perpetual stasis, reaching $\bar{t_{R}}≳11\;t_{c}$ over 20 cycles of simulation, did not respond to 97.5% factor XI inhibition but did respond to a similar inhibition of factor XII. These regions of nearly quasi-perpetual stasis were found in all the *high prothrombotic risk* cases. Particularly, subject 11 had areas with $\bar{t_{R}}\geq 14t_{c}$ after 20 simulation cycles and had LAA thrombin concentrations very close to the coagulating threshold even after a 97.5% inhibition of factor XII (Fig. 12D).

## 4. Discussion

The relevance of left heart flow patterns on thrombosis and cardiogenic stroke has been recognized for decades [60–62]. Recent clinical studies have further solidified the causal association between intracardiac stasis and brain embolism [63–65]. Modeling cardiac thrombosis in vivo is particularly challenging, as coagulation times measured in humans differ significantly from those measured in commonly used large animal models (e.g., calves, sheep, goats, and pigs) [66]. Although computational models offer an alternative, their high computational cost has limited their use. Despite noteworthy pioneering efforts using idealized models [67–70] and patient-specific anatomies [71,72], there are no systematic simulation studies of intracardiac coagulation considering different clinical scenarios or individual responses to different anticoagulantion regimes. To address this gap, we developed efficient multi-fidelity coagulation cascade models and conducted a analyzing the interplay between blood flow, coagulation and factor XI/XII inhibition in 3D patient-specific anatomies.

### Multi-fidelity modeling enables realistic coagulation analysis in patient-specific anatomies

Mathematical models of the coagulation cascade are often formulated as systems of ordinary differential equations (ODEs) representing the cascade’s reaction kinetics [44]. These ODE systems are valid when the coagulation components form a homogeneous mixture in the volume of interest, but intracardiac flow creates regions with different transport profiles that sometimes impede homogeneous mixing [73,74]. High-fidelity (HiFi) models of intracardiac coagulation involve 3D advection–diffusion–reaction partial differential equations (PDEs). Solving these PDEs is computationally intensive, given the number of coagulation components involved and their multi-scale nature.

Multi-fidelity (MuFi) coagulation modeling is based on the observation that the reaction terms in the PDEs governing components concentration can be evaluated independently at each spatial point as long as there is no mass diffusivity, so that these equations can be converted into ODEs [75]. This idea has been recently formalized and extended to non-zero diffusivities by Taylor-expanding the ODEs around the zero-diffusivity limit, producing spatiotemporal maps of component concentrations in terms of the statistical moments of residence time [23]. The MuFi approach reduces the problem of solving N PDEs for N coagulation components to p PDEs for the first p statistical moments of $\bar{t_{R}}$ and N ODEs for the reaction kinetics. Therefore, by reproducing the HiFi results for a given cardiovascular geometry, reaction kinetics, and sufficiently low order p, MuFi modeling accelerates blood coagulation simulations under flow.

In this work, the effectiveness of the MuFi models was verified in patient-derived LA flows by comparing them to the HiFi reference model for the 9-species coagulation system with reaction kinetics described in [58]. Due to the elevated computational cost of the HiFi simulations, we restricted the verification to two distinct patient-derived LA flows: one corresponding to a subject in sinus rhythm with normal LA function and another with AF and impaired LA function. In both cases, a second-order MuFi model (MuFi-2) captured the spatiotemporal thrombin dynamics over 20 cardiac cycles with less than 10% errors.

MuFi models accelerate the simulations of the coagulation cascade under flow in two ways. First, running one instance of a given coagulation model is predicted to be αN/p times faster in MuFi-p form than in HiFi form, where α≳1 is a proportionality constant [23]. Second, and more important, MuFi models decouple the CFD and the coagulation solvers, providing countless virtually free coagulation cascade simulations per CFD run. Therefore, running a series of k coagulation cascade simulations on one patient can achieve a speedup of ~kαN/p, which grows boundlessly with the size of the campaign and the number of coagulation components. As an example, let us consider the 32-component model used for the MuFi-2 simulation campaign in this study. Extrapolating the speedup values of 7.7 and 4.4 obtained running MuFi-2 and MuFi-3 for the 9-component model [58], αN/p≈25 is a reasonable estimate for the 32-component model. Then, since each inhibition study reported in this manuscript involved k=19 MuFi-2 coagulation simulations (no inhibition plus 9 inhibitions of Factors XI and XII), the cumulative speedup of the entire simulation campaign would be ≈ 19 × 25 = 475. It is difficult to imagine that the simulations presented in this manuscript would be feasible if they required 475 times more computational time.

### Left atrial coagulation under patient-derived flow

Our data support the hypothesis that flow patterns in the LA, particularly in the LAA (e.g., slow velocities, long residence time), play a crucial role in thrombosis. This view is strengthened by clinical data associating LAA blood stasis and increasing time in AF with higher thromboembolic risk [76]. The consensus emerging from recent simulation studies is that LAA blood stasis depends on multiple factors including LA function [48], position, orientation and/or flow split of the pulmonary veins [57,77–79], and LAA morphology [80–83]. The residence time of blood inside the left atrium is often used as a surrogate metric for thrombosis risk. Many studies report that $\bar{t_{R}}$ peaks inside the LAA [77,79,84–95].

In our simulations, the concentration of thrombin peaked inside the LAA but its temporal dynamics were significantly more complex than those of $\bar{t_{R}}$ due to the multi-scale nature of coagulation cascade kinetics. Based on these dynamics, we identified 3 groups of patients. One group – that we defined as having *high prothrombotic risk* – showed exponential growth of the thrombin concentrations. A second group (*moderate prothrombotic risk*) showed more intricate dynamics with significantly slower growth that did not surpass the coagulation threshold. Finally, a third group showed normal flow dynamics and no thrombin accumulation. Thus, we classified this group it as having *no prothrombotic risk*.

Although our small sample size prevented a rigorous statistical analysis of our models’ predictive value, we observed a relationship between the thrombotic risk of the patients, their LAA flow pattern, and clinical embolism risk factors like AF, LAA volume, and $EF_{LA}$. All *high prothrombotic risk* cases were studied in AF and had severely impaired atrial function (emptying fraction $F_{LA}≲0.13$) associated with significant blood stasis in the LAA (${\left\langle \bar{t_{R}}\right\rangle }_{\text{LAA}}/t_{c}\approx 5.18\pm 1.26$). Conversely, most of the *no prothrombotic risk* cases involved patients in sinus rhythm, with relatively normal LA function, low LAA stasis $\left({\left\langle \bar{t_{R}}\right\rangle }_{\text{LAA}}/t_{c}\approx 2.12\pm 0.33\right)$, and no prior thromboembolic events. This group even included a patient (Case 10) with prior AF but reverted to sinus rhythm that had the highest $EF_{LA}$ among the AF group $\left(EF_{LA}=\right.0.26$). The cases with *moderate prothrombotic risk* had mixed risk factors. One had a normal atrial function but a relatively large LAA, while the others were imaged in AF and had moderately impaired atrial function $\left(EF_{LA}\approx 0.23\right)$. LAA residence time in the *moderate-risk* group $\left({\left\langle \bar{t_{R}}\right\rangle }_{\text{LAA}}/t_{c}\approx 3.61\pm 1.45\right)$, was between the *no-risk* and the *high-risk* groups. Of note, all the patients with history of thromboembolic events (LAA thrombus or TIA) had hemodynamic substrates associated with *moderate* or *high prothrombotic risk*.

### The efficacy of factor XI/XII inhibition depends on patient-derived LAA flow

Selecting the anticoagulation treatment and dosage for individual patients with AF is a complex decision that needs to balance the treatment’s decrease in thrombosis risk with its increase of hemorrhagic risk [5,96]. DOACs are preferred over anti-vitamin K inhibitors for their more predictable pharmacokinetics. However, dose regimens of current DOACs are based on standardized dose–response curves derived from in vitro laboratory assays [97]. Anticoagulant agents targeting the intrinsic pathway, such as factor XI inhibitors, are promising due to their potential to reduce bleeding risks, as evidenced by recent phase-II randomized clinical trials [16]. However, the need for premature interruptions in some phase III studies [17] suggests the dose–response relationship of each DOAC drug must be understood under realistic situations mimicking their *in vivo* applications. *In vitro* assays may be insufficient for this purpose because they do not reproduce each individual’s particularities of LA flow transport. Our simulations of factor XI and factor XII inhibition in 13 patient-derived LA models suggest that no-flow models overpredict thrombin concentration, underscoring the importance of accounting for patient-specific flow patterns when studying LA anticoagulation.

We observed that inhibition of factor XI and, particularly, factor XII reduced thrombin growth in our patient-derived models. *In vitro* and in vivo experiments [15,98–100] suggest that inhibition levels between 70% and 90% can significantly lower thrombin concentrations, in agreement with our results. Moreover, Heitmeier et al. [99] observed a 3-fold increase in the activated partial thromboplastin time (aPTT) after inhibiting the activation of factor XI (asundexian). This result is in line with the 1.5 fold increase in $t_{co}$ reported in Fig. 11A, even if physiological values of aPTT are in the range of 20 to 30s, considerably longer than the values of $t_{co}$ in our study. Being consistent with previous studies, our results also demonstrate that the inhibition required to prevent thrombosis in the LA depends on intra-atrial flow patterns. Some (*moderate-risk*) cases required only modest inhibition (<50%) to prevent thrombin growth. However, other (*high-risk*) cases required nearly complete inhibition (97.5%) of factor XII to stop thrombin growth. In these patients, inhibition of factor XI proved insufficient at any dose, consistent with reported outcomes from clinical trials of the factor XI inhibitor asundexian [18]. Since we used the same reaction kinetics for all patients, the observed differences in coagulation dynamics and anticoagulant efficacy must be attributed to differences in LA mechanical and fluid dynamic properties. In particular, we related these differences to the spatiotemporal distributions of the residence time inside the LAA.

### Clinical implications

Current doses of DOACs for stroke prevention in AF tested in the pivotal randomized clinical trials are adjusted mostly based on pharmacokinetic factors (i.e: patient weight, renal function) [9–12]. Our findings suggest that anticoagulant regimens may also need be adjusted according to idiosyncratic anatomical and functional characteristics. Further research is needed to clarify whether anticoagulation efficacy can be inferred from indirect features easier to obtain in the clinical setting such as clinical or anatomic data. However, meanwhile, current and future drugs may benefit of being tested *in silico* to ensure efficiency. The observation that current DOAC regimens may be inadequate in patients with highly stagnant LAAs may explain the clinical observations of recurrent embolic events in AF patients despite “appropriate” anticoagulation [101]. In such cases, LAA occlusion might be a preferable treatment strategy.

Computationally efficient tools, such as the MuFi model introduced here, facilitate cost-effective analysis of diverse hemodynamic scenarios. By integrating medical imaging, flow analysis, and individualized coagulation factor evaluation, these tools support personalized risk assessment and anticoagulation therapy optimization. These tools could also refine patient selection for clinical trials of novel anticoagulation drugs.

### Study limitations

This study’s patient group, N=13, is significantly larger than the N=2 used in the only other LA clotting simulation study we know of Qureshi et al. [72]. However, it is still far from sufficient to achieve statistical power. Moreover, our patient selection prioritized achieving a wide range of atrial functions and volumes and over-represented AF and LAA thrombosis to demonstrate the interplays between AF, LA hemodynamics, and thrombosis. For these reasons, although we found interesting trends between LAA coagulation species concentration (no, moderate and *high prothrombotic risk*) and patient’s clinical data (AF, presence of LAA thrombus or prior TIA), the data was insufficient to confirm a correlation.

We note but are less concerned about the subjectivity of classifying patients as *non-coagulating*, *moderately coagulating*, or *severely coagulating*. Given that the definition of coagulation time is not unique and the thrombin threshold values used to identify clotting vary in the range of 2–15 nM [25,42,102], our classification may seem somewhat subjective (exponential growth vs. significantly slower growth; maximum thrombin concentration over threshold value $u_{IIa}^{th}$), but our data suggest it is reasonably robust. If we lowered or raised this threshold concentration by a factor of 2, only one case would switch class between *non-coagulating* to *moderately coagulating*. And changing the definition of sufficiently fast growth for the *severely coagulating* group might switch only one case from *moderately* to *severely coagulating*, if any.

Although our CFD simulations used personalized LA shapes and motion obtained from 4D-CT imaging, several parameters in our models were not patient-specific. Below, we discuss these parameters and the potential limitations of using generic values across the patient population. In all cases, the principal reason for using generic values was the lack of data either in direct form or in a form that would allow for identifying parameters in our models. Considering this lack and that our main objective was to evaluate the effect of factor XI/XII inhibition on LA thrombosis, it seemed sound to systematically vary the initial concentrations of factor XI/XII while keeping all other parameters constant. This approach has allowed for a consistent comparison of metrics across different patients and facilitated a systematic evaluation of the effect of factor XI/XII inhibition on a specific patient.

All simulations were run at a constant heart rate (60 bpm), and the PV flow rates were evenly split to set inflow/outflow boundary conditions based on each patient’s LV and LA time-dependent volumes, as in [48]. While prior studies justify this choice [103], the PV flow split could affect flow patterns inside the LAA, particularly in cases where LAA residence time is high and thrombosis is more likely [57]. We considered non-Newtonian blood rheology, but we fixed the hematocrit value (Hct=43.5) because this parameter was unavailable for some patients. We also fixed the characteristic time of RBC aggregation $\left(t_{\mu }=3\;\text{s}\right)$ as described in [50]. These two parameters could impact residence time and non-Newtonian effects in the LA [104]. The reaction kinetics in the coagulation cascade model were not patient-specific either, using the same reaction rates and initial conditions for all cases. Variations in the central blood concentration of coagulation factors and equilibrium constants can be significant [105], which would affect the time evolution of the maximum thrombin concentration in the LAA. We did not have patient-specific measurements of these concentrations or clotting times, and even if these data were available, many parameters of our detailed 32-species kinetic model would not be identifiable in a patient-specific manner.

We only considered the intrinsic coagulation pathway since our main focus was factor XI/XII inhibition, ignoring contributions from the extrinsic pathway (including endothelial effects) that could influence thrombin generation [8]. Due to the high computational cost of running HiFi simulations in 3D patient-specific anatomies, we verified our MuFi approach vs. the HiFi approach using a 9-species model [58], then ran our simulation campaign using a 32-species model [24]. The coagulation model in our work refers exclusively to the simulation of the intrinsic coagulation cascade in conjunction with CFD. We do not include any model accounting for thrombus formation and dynamics, like Zheng et al. [106] and Xu et al. [107]. Expanding our framework to include thrombus formation is left for future work.

Finally, we adopted an oversimplistic representation of anticoagulation therapy by directly varying the inhibition level of each target factor (${\text{INH}}_{i}$). In clinical practice, only anticoagulant dosages can be modified or adapted based on patient-specific clinical characteristics, but controlling ${\text{INH}}_{i}$ is more challenging due to numerous factors interfering with their effect such as drug absorption, distribution, metabolism, and elimination [108].

## 5. Conclusions

We applied multi-fidelity (MuFi) coagulation cascade modeling in the low molecular diffusivity limit to 3D patient-specific left atrial segmentations with hemodynamics obtained from computational fluid dynamics. MuFi schemes couple flow-mediated transport and reaction kinetics via p residence-time-like variables representing the scheme’s order. We demonstrated that MuFi models of order p≥2 accurately capture the spatiotemporal dynamics of coagulation species concentrations in the LAA of 3D patient-specific models, accelerating simulations by over two orders of magnitude. This computational efficiency enabled an extensive study of 247 simulations, which to the best of our knowledge, constitutes the first systematic investigation of intracardiac coagulation and anticoagulation therapy using 3D patient-specific anatomies and hemodynamics.

We considered a detailed 32-species model of the intrinsic coagulation pathway, systematically varying factor XI/XII inhibition levels across 13 patient-specific left atria with diverse anatomical and functional characteristics, including cases of sinus rhythm, AF, and left atrial appendage thrombosis. Our findings indicate that thrombin exhibited the most significant growth in the LAA of patients with impaired blood washout, particularly in those with AF and poor left atrial function. These cases, which we classified as *high prothrombotic risk*, exhibited explosive growth of thrombin, while cases of *moderate prothrombotic risk* showed slower accumulation of thrombin. Cases with normal atrial function and sinus rhythm experienced minimal thrombin production inside the LAA throughout the simulations. Furthermore, we found that high risk cases required significantly more aggressive factor XI/XII inhibition to arrest thrombin growth compared to *moderate prothrombotic risk* ones, underscoring the role of patient-specific hemodynamics in determining anticoagulation response.

These findings suggest that the effectiveness of novel anticoagulation agents targeting the intrinsic coagulation pathway in AF may strongly depend on patient-specific flow patterns. By providing computationally efficient tools to study this dependence, this work lays the foundation for the in-silico determination of personalized dose–response curves for DOACs and the optimization of patient selection for clinical trials. Additionally, our approach highlights the potential for leveraging medical imaging-based patient-specific modeling to personalize anticoagulation therapy, offering a pathway toward precision medicine in thromboembolic disease prevention.

## Figures and Tables

**Table 1:** Nominal initial concentrations.

Factor	Concentration [μM]	Reference
PK	0.58	Saito et al. [26]
XIIa	$2.3\cdot 10^{-5}$	Zhu [24]
C1	1.7	Harpel [27]
PAI	$4.6\cdot 10^{-4}$	Kruithof et al. [28]
$\alpha_{2}\text{M}$	3.5	Harpel [27]
ATIII	3.4	Collen et al. [29]
XII	0.6	Madsen et al. [30]
XI	0.06	Gailani and Broze [31]
$\alpha_{2}\text{AP}$	0.9	Harpel [27]
IX	0.18	Komiyama et al. [32]
X	0.34	Tormoen et al. [33]
TFPI	$2.5\cdot 10^{-3}$	Novotny et al. [34]
$\alpha_{1}\text{AT}$	24.5	Harpel [27]
IIa	$2\cdot 10^{-4}$	–
II	1.8	Monroe et al. [35]
V	0.042	Tracy et al. [36]
TM	$2.2\cdot 10^{-4}$	Aso et al. [37]
PC	0.064	Vaziri et al. [38]
VIII	$1.4\cdot 10^{-3}$	Butenas et al. [39]
I	8.3	Ratnoff and Menzie [40]

**Table 2:** Clinical data and anatomical/functional parameters of the LA and the LAA. The mean volume values represent time-averaged volumes. The emptying fraction for LA and LAA are defined as $EF_{LA}=\left(\text{max}\left(V_{LA}\right)-\text{min}\left(V_{LA}\right)\right)/\text{max}\left(V_{LA}\right)$ and $EF_{LAA}=\left(\text{max}\left(V_{LAA}\right)-\text{min}\left(V_{LAA}\right)\right)/\text{max}\left(V_{LAA}\right)$, respectively. TIA stands for Transient Ischemic Attacks. The mean LAA residence time ${\left\langle \bar{t_{R}}\right\rangle }_{\text{LAA}}$ is computed averaging in space and time during the 20th cycle of the CFD simulations.

Subject	1	2	3	4	5	6	7	8	9	10	11	12	13	Avg	Std
Age	40	62	65	82	92	50	79	80	83	58	91	61	54	–	-
Sex	M	M	F	M	F	M	F	F	F	M	M	M	F	–	-
${\text{CHA}}_{2}{\text{DS}}_{2}\text{-VASc}$	–	–	–	6	6	1	4	2	3	1	6	2	4	–	-
LAA thrombus	No	No	No	TIA	Yes	Yes	No	No	No	No	Yes	No	No	–	–
Persistent AF	No	No	No	No	Yes	Yes	Yes	Yes	Yes	Yes	Yes	No	No	–	–
Sinus rhythm	Yes	Yes	Yes	Yes	No	No	No	No	No	Yes	No	Yes	Yes	–	–
Mean LA vol, (ml)	86.6	70.1	115	145	157	180	229	177	193	132	150	84	96	139.6	47.9
Max LA vol, (ml)	108	91.2	145	155	165	205	247	194	208	157	160	108	121	158.8	45.6
Min LA vol, (ml)	59.6	49.0	87.2	119	150	157	216	169	183	116	139	60.3	68.6	121	53.4
Mean LAA vol, (ml)	6.94	4.85	14.3	10.7	15.5	22.0	5.51	6.17	14.1	14.1	15.7	3.13	3.64	10.5	5.9
Max LAA vol, (ml)	8.97	6.28	17.9	11.6	17.4	24.7	6.58	7.41	15.47	17.0	18.5	4.23	4.65	12.4	6.5
Min LAA vol, (ml)	4.32	3.14	10.2	9.10	13.8	19.8	5.01	5.17	13.4	12.5	13.16	1.45	2.24	8.7	5.6
$EF_{LA}$	0.45	0.46	0.4	0.23	0.09	0.23	0.123	0.127	0.12	0.26	0.132	0.44	0.43	0.27	0.15
$EF_{LAA}$	0.52	0.50	0.43	0.2	0.21	0.22	0.24	0.30	0.13	0.26	0.28	0.66	0.52	0.34	0.16
Anticoagulants	No	No	No	No	Yes	Yes	Yes	Yes	Yes	Yes	Yes	No	No	–	–
${\left\langle \bar{t_{R}}\right\rangle }_{\text{LAA}}/t_{c}$	2.59	1.84	2.01	3.98	4.83	3.71	7.19	5.85	5.30	2.20	5.84	1.78	2.23	3.8	1.8

## Appendix

### A.1. Coagulation cascade for the verification runs.

In this section, we describe the reaction terms for the 9-species coagulation model employed in the validation study. The model, initially introduced by Zarnitsina et al. [58], incorporates three positive feedback loops, which are facilitated by thrombin-activated factors XIa, Va, and VIIIa. Additionally, it includes negative feedback mechanisms, where factors Va and VIIIa are deactivated by the generation of PCa, which is itself activated by thrombin. The interactions of these feedback loops are schematically illustrated in Fig. 1 of Zarnitsina et al. [58]. The model was validated against experiments by Ataullakhanov et al. [109] for various calcium concentrations. The reaction terms for the transport equations of the 9 species are given in Eqs. (18)–(27), and the reaction coefficients reported by Ataullakhanov et al. [109] are listed in Table 4. The same diffusivity is assumed for all species, with $D=10^{-7}{\;\text{cm}}^{2}/\text{s}$.

(18)
$$R_{XIa}=k_{11}\left[IIa\right]-h_{11}\left[XIa\right],$$

(19)
$$R_{IXa}=k_{9}\left[XIa\right]-h_{9}\left[IXa\right],$$

(20)
$$R_{Xa}=k_{10}\left[IXa\right]+\bar{k_{10}}\frac{k_{89}\left[IXa\right]\left[\text{VIIIa}\right]}{h_{89}+k_{a}\left[PCa\right]}-h_{10}\left[Xa\right],$$

(21)
$$R_{IIa}=k_{2}\left[Xa\right]\frac{\left[II\right]}{\left[II\right]+K_{2m}}+\bar{k_{2}}\frac{k_{510}\left[Xa\right]\left[Va\right]}{h_{510}+k_{a}\left[PCa\right]}\frac{\left[II\right]}{\left[II\right]+\bar{K_{2m}}}-h_{2}\left[IIa\right],$$

(22)
$$R_{II}=-k_{2}\left[Xa\right]\frac{\left[II\right]}{\left[II\right]+K_{2m}}-\bar{k_{2}}\frac{k_{510}\left[Xa\right]\left[Va\right]}{h_{510}+k_{a}\left[PCa\right]}\frac{\left[II\right]}{\left[II\right]+\bar{K_{2m}}},$$

(23)
$$R_{\text{VIIIa}}=k_{8}\left[IIa\right]-k_{a}\left[PCa\right]\left(\left[\text{VIIIa}\right]+\frac{k_{89}\left[IXa\right]\left[\text{VIIIa}\right]}{h_{89}+k_{a}\left[PCa\right]}\right)-h_{8}\left[\text{VIIIa}\right],$$

(24)
$$R_{Va}=k_{5}\left[IIa\right]-k_{a}\left[PCa\right]\left(\left[Va\right]+\frac{k_{510}\left[Xa\right]\left[Va\right]}{h_{510}+k_{a}\left[PCa\right]}-h_{5}\left[Va\right]\right),$$

(25)
$$R_{PCa}=\frac{k_{apc1}k_{p}+k_{apc2}P}{k_{p}+P}[IIa]-h_{apc}[PCa],$$

(26)
$$R_{Ia}=k_{11}[IIa],$$

(27)
$$P=\frac{\left(k_{apc2}[IIa]-h_{p}k_{p}\right)+\sqrt{{\left(k_{apc2}[IIa]-h_{p}k_{p}\right)}^{2}+4k_{apc1}k_{p}h_{p}[IIa]}}{2h_{p}}.$$

**Table 3:** Initial conditions.

Species concentrations	Initial condition [nM]
$u_{XIa}$	0.105
$u_{IXa}$	11.024
$u_{Xa}$	0.202
$u_{\text{IIa}\;}$	92.626
$u_{II}$	867.564
$u_{\text{VIIIa}}$	$1.534\cdot 10^{-4}$
$u_{Va}$	2.713
$u_{\text{PCa}\;}$	$3.488\cdot 10^{-2}$
$u_{Ia}$	48.811

**Table 4:** Reaction rates.

Coeff.	Value	Coeff.	Value	Coeff.	Value
$k_{1}$	$2.82{\;\text{min}}^{-1}$	$k_{2}$	$2.45{\;\text{min}}^{-1}$	$\bar{k_{2}}$	$2\cdot 10^{3}{\;\text{min}}^{-1}$
$h_{2}$	$2.3{\;\text{min}}^{-1}$	$k_{5}$	$0.17{\;\text{min}}^{-1}$	$h_{5}$	$0.31{\;\text{min}}^{-1}$
$k_{8}$	$1\cdot 10^{-5}{\;\text{min}}^{-1}$	$h_{8}$	$0.31{\;\text{min}}^{-1}$	$k_{9}$	$20{\;\text{min}}^{-1}$
$h_{9}$	$0.2{\;\text{min}}^{-1}$	$k_{10}$	$3.3\cdot 10^{-3}{\;\text{min}}^{-1}$	$\bar{k_{10}}$	$500{\;\text{min}}^{-1}$
$h_{10}$	$1{\;\text{min}}^{-1}$	$k_{11}$	$1.1\cdot 10^{-5}{\;\text{min}}^{-1}$	$h_{11}$	$0.2{\;\text{min}}^{-1}$
$k_{89}$	$100(\text{nM min}{)}^{-1}$	$h_{89}$	$100{\;\text{min}}^{-1}$	$k_{510}$	$100(\text{nM min}{)}^{-1}$
$h_{510}$	$100{\;\text{min}}^{-1}$	$h_{\text{apc}}$	$0.1{\;\text{min}}^{-1}$	$k_{\text{apc}1}$	$1.4\cdot 10^{-3}{\;\text{min}}^{-1}$
$k_{apc2}$	$7\cdot 10^{-2}{\;\text{min}}^{-1}$	$k_{a}$	$1.2(\text{Mn min}{)}^{-1}$	$k_{p}$	10 nM
$h_{p}$	$1{\;\text{min}}^{-1}$	$K_{2m}$	58 nM	$\bar{K_{2m}}$	210 nM

**Table 5:** Enzymatic reactions, and corresponding kinetic parameters. K, kallikrein; PK, prekallikrein; PL, phospholipids; TM, thrombomodulin. All reactions are Michaelis–Menten reactions except the last one, which is a first order reaction with $k_{55}=0.0078{\;\text{min}}^{-1}$.

Reaction	$k_{\text{cat}}\;\left({\text{min}}^{-1}\right)$	$k_{m}\;(\mu \text{mol}/\text{l})$
$XII\overset{\text{XIIa}}{\to }\text{XIIa}$	1.98	11
$PK\overset{\text{XIIa}}{\to }K$	216	0.091
$PK\overset{XII_{f}}{\to }K$	2400	37
$XII\overset{K}{\to }{XII}_{a}$	342	0.51
$XII_{a}\overset{K}{\to }XII_{f}$	0.34	0.5
$XI\overset{XII_{a}}{\to }XI_{a}$	0.034	2
$XII\overset{XI_{a}}{\to }XII_{a}$	34	0.5
$IX\overset{XI_{a}}{\to }IX_{a}$	225	0.35
$X\overset{IX_{a}}{\to }Xa$	0.04	2.0
$X\overset{IX_{a}-VIII_{a}-PL}{\to }Xa$	1740	0.19
$II\overset{Xa}{\to }IIa$	2.25	0.058
$II\overset{Xa-Va-PL}{\to }IIa$	1700	1.0
$V\overset{IIa}{\to }Va$	14	0.0717
$V\overset{Xa}{\to }Va$	2.6	0.0104
$\text{VIII}\overset{\text{IIa}}{\to }\text{VIIIa}$	60	0.02
$I\overset{IIa}{\to }Ia$	5040	7.2
$PC\overset{IIa-TM}{\to }PCa$	19.8	7.7
$XI\overset{\text{IIa}}{\to }XIa$	–	–

### A.2. Coagulation cascade model for initiation and inhibition study.

In this section, we present the 32-species coagulation model used in the coagulation risk and inhibition study. This model comprises the intrinsic pathway of the coagulation cascade, and it is thoroughly described and analyzed in [24]. A schematic representation of the model can be found in Fig. 1 of Zhu [24]. The chemical reactions and their corresponding kinetic constants are summarized in Tables 5 and 6. The reaction term for the transport equation of each species is given by

(28)
$$R_{K}=\frac{k_{\text{cat}2}\left[XII_{a}\right][PK]}{k_{m2}+[PK]}+\frac{k_{\text{cat}3}\left[XII_{f}\right][PK]}{k_{m3}+[PK]}-k_{39}\left[C1\right]\left[K\right]-k_{40}\left[\alpha_{2}M\right]\left[K\right]-k_{41}\left[PAI-1\right]\left[K\right]-k_{42}\left[AT-III\right]\left[K\right];$$

(29)
$$R_{PC_{a}}=\frac{k_{\text{cat}54}[PC][\text{IIaTM}]}{k_{m54}+[PC]}-\left[PC_{a}\right]\left(k_{49}\left[V_{a}\right]+k_{50}\left[VIII_{a}\right]\right.\left.+k_{51}\left[V_{a}X_{a}\right]+k_{52}\left[VIII_{a}IX_{a}\right]\right);$$

(30)
$$R_{PC}=-\frac{k_{\text{cat}54}[PC][\text{IIaTM}]}{k_{m54}+[PC]};$$

(31)
$$R_{I_{a}}=\frac{k_{\text{cat}16}[IIa][I]}{k_{m16}+[I]};$$

(32)
$$R_{PK}=-\frac{k_{\text{cat}2}\left[XII_{a}\right][PK]}{k_{m2}+[PK]}-\frac{k_{\text{cat}3}\left[XII_{f}\right][PK]}{k_{m3}+[PK]};$$

(33)
$$R_{\text{XIIa}}=\frac{k_{\text{cat}4}[XII][K]}{k_{m4}+[XII]}+\frac{k_{\text{cat}7}[XIa][XII]}{k_{m7}+[XII]}+\frac{k_{\text{cat}1}[XII]\left[XII_{a}\right]}{k_{m1}+[XII]}-\frac{k_{\text{cat}5}\left[XII_{a}\right][K]}{k_{m5}+\left[XII_{a}\right]}-k_{31}[C1]\left[XII_{a}\right]-k_{32}\left[\alpha_{2}AP\right]\left[XII_{a}\right]-k_{33}\left[\alpha_{2}M\right]\left[XII_{a}\right]-k_{34}[AT-III]\left[XII_{a}\right]-k_{35}[PAI-1]\left[XII_{a}\right];$$

(34)
$$R_{XII_{f}}=\frac{k_{\text{cat}}\left[XII_{a}\right][K]}{k_{m5}+\left[XII_{a}\right]}-k_{36}[C1]\left[XII_{f}\right]-k_{37}\left[\alpha_{2}AP\right]\left[XII_{f}\right]-k_{38}[AT-III]\left[XII_{f}\right];$$

(35)
$$R_{XII}=-\frac{k_{\text{cat}4}\left[XII\right]\left[K\right]}{k_{m4}+\left[XII\right]}-\frac{k_{\text{cat}7}\left[XIa\right]\left[XII\right]}{k_{m7}+\left[XII\right]}-\frac{k_{\text{cat}1}\left[XII\right]\left[XII_{a}\right]}{k_{m1}+\left[XII\right]};$$

(36)
$$R_{XIa}=\frac{k_{\text{cat}6}\left[XII_{a}\right][XI]}{k_{m6}+[XI]}+k_{55}[XI]-k_{26}[C1][XIa]-k_{27}\left[\alpha_{1}AT\right][XIa]-k_{28}[AT-III][XIa]-k_{29}\left[\alpha_{2}AP\right][XIa]-k_{30}[PAI-1][XIa];$$

(37)
$$RXI=-\frac{k_{\text{cat}6}\left[XII_{a}\right][XI]}{k_{m6}+[XI]}-k_{55}[XI];$$

(38)
$$R_{C1}=-[C1]\left(k_{39}[K]+k_{31}\left[XII_{a}\right]+k_{26}[XIa]+k_{36}\left[XII_{f}\right]\right);$$

(39)
$$R_{\alpha_{2}M}=-\left[\alpha_{2}M\right]\left(k_{40}[K]+k_{33}\left[XII_{a}\right]\right);$$

(40)
$$R_{PAI-1}=-[PAI-1]\left(k_{41}[K]+k_{35}\left[XII_{a}\right]+k_{30}[XIa]\right);$$

(41)
$$R_{AT-III}=-[AT-III]\left(k_{42}[K]+k_{34}\left[XII_{a}\right]+k_{38}\left[XII_{f}\right]+k_{28}[XIa]\right);$$

(42)
$$R_{\alpha_{2}AP}=-\left[\alpha_{2}AP\right]\left(k_{32}\left[XII_{a}\right]+k_{37}\left[XII_{f}\right]+k_{29}[XIa]\right);$$

(43)
$$R_{\alpha_{1}AT}=-k_{27}\left[\alpha_{2}AP\right][XIa];$$

(44)
$$R_{IX}=-\frac{k_{cat8}[XIa][IX]}{k_{m8}+[IX]};$$

(45)
$$R_{IXa}=\frac{k_{\text{cat}8}[XIa][IX]}{k_{m8}+[IX]}-k_{18}\left[VIII_{a}\right][XIa]-k_{25}[AT-III][XIa];$$

(46)
$$R_{X}=-\frac{k_{\text{cat}}[XIa][X]}{k_{m9}+[X]}-\frac{k_{\text{cat}10}\left[\text{XIaVI}I_{a}\right][X]}{k_{m10}+[X]};$$

(47)
$$R_{Xa}=\frac{k_{\text{cat}9}[XIa][X]}{k_{m9}+[X]}+\frac{k_{\text{cat}10}\left[\text{XIaVII}I_{a}\right][X]}{k_{m10}+[X]}-k_{17}\left[V_{a}\right]\left[X_{a}\right]-k_{22}[AT-III]\left[X_{a}\right]-k_{24}[\text{TFPI}]\left[X_{a}\right]-k_{23}\left[\alpha_{1}AT\right]\left[XI_{a}\right];$$

(48)
$$R_{\text{XaVII}I_{a}=}k_{18}\left[X_{a}\right]\left[VIII_{a}\right];$$

(49)
$$R_{II}=-\frac{k_{cat11}\left[X_{a}\right]\left[II\right]}{k_{m11}+\left[II\right]}-\frac{k_{cat12}\left[V_{a}X_{a}\right]\left[II\right]}{k_{m12}+\left[II\right]};$$

(50)
$$R_{IIa}=\frac{k_{cat11}\left[X_{a}\right][II]}{k_{m11}+[II]}+\frac{k_{cat12}\left[V_{a}X_{a}\right][II]}{k_{m12}+[II]}-k_{19}\left[AT-III\right]\left[IIa\right]-k_{21}\left[\alpha_{2}M\right]\left[X_{a}\right]-k_{20}\left[\alpha_{1}AT\right]\left[IIa\right]-k_{53}\left[IIa\right]\left[TM\right];$$

(51)
$$R_{V}=-\frac{k_{cat13}\left[IIa\right]\left[V\right]}{k_{m13}+\left[V\right]}-\frac{k_{cat14}\left[X_{a}\right]\left[V\right]}{k_{m14}+\left[V\right]};$$

(52)
$$R_{V_{a}}=\frac{k_{cat13}[IIa][V]}{k_{m13}+[V]}+\frac{k_{cat14}\left[X_{a}\right][V]}{k_{m14}+[V]}-k_{17}\left[V_{a}\right]\left[X_{a}\right]-k_{49}\left[V_{a}\right]\left[PC_{a}\right];$$

(53)
$$R_{\text{VIII}}=-\frac{k_{cat15}\left[IIa\right]\left[VII\right]}{k_{m15}+\left[\text{VIII}\right]};$$

(54)
$$R_{VIII_{a}}=\frac{k_{cat15}[IIa][\text{VIII}]}{k_{m15}+[\text{VIII}]}-k_{18}\left[VIII_{a}\right]\left[X_{a}\right]-k_{50}\left[VIII_{a}\right]\left[PC_{a}\right];$$

(55)
$$R_{V_{a}X_{a}}=k_{17}\left[V_{a}\right]\left[X_{a}\right]-k_{51}\left[V_{a}X_{a}\right]\left[PC_{a}\right];$$

(56)
$$R_{TM}=-k_{53}\left[TM\right]\left[IIa\right];$$

(57)
$$R_{\text{IIaTM}}=k_{53}[TM][IIa];$$

(58)
$$R_{\text{TFPI}}=-k_{24}[\text{TFPI}]\left[X_{a}\right];$$

(59)
$$R_{I}=-\frac{k_{cat16}\left[IIa\right]\left[I\right]}{k_{m16}+\left[I\right]}.$$

**Table 6:** Second-order reactions and corresponding kinematic parameters. $\alpha_{2}AP,\alpha_{2}$-antiplasmin; $\alpha_{1}AT,\alpha_{1}$-antitrypsin; AT-III, antithrombin III; C1, C1-inhibitor; $\alpha_{2}M,\alpha_{2}$-macroglobulin; PAI-1, plasminogen activator inhibitor-1; PC, protein C; TFPI, tissue factor pathway inhibitor; TM, thrombomodulin.

Reaction	k(μmol/l per min)
Va+Xa→Va-Xa	$10^{4}$
VIIIa+IXa→VIIIa-IXa	$10^{4}$
IIa+AT-III→IIa-AT-III	0.35
$\text{IIa}+\alpha_{1}\text{AT}\to \text{IIa}-\alpha_{1}\text{AT}$	0.0047
$\text{IIa}+\alpha_{2}\text{M}\to \text{IIa}-\alpha_{2}\text{M}$	0.0293
Xa+AT-III→Xa-AT-III	0.11
$\text{Xa}+\alpha_{1}\text{AT}\to \text{Xa}-\alpha_{1}\text{AT}$	0.0157
Xa+TFPI→Xa-TFPI	960
IXa+AT-III→IXa-AT-III	0.0294
XIa+C1→XIa-C1	0.001
$\text{XIa}+\alpha_{1}\text{AT}\to \text{XIa}-\alpha_{1}\text{AT}$	0.004
XIa+AT-III→XIa-AT-III	0.01
$\text{XIa}+\alpha_{2}\text{AP}\to \text{XIa}-\alpha_{2}\text{AP}$	0.03
XIa+PAI-1→XIa-PAI-1	12.6
XIIa+C1→XIIa-C1	0.22
$\text{XIIa}+\alpha_{2}\text{AP}\to \text{XIIa}-\alpha_{2}\text{AP}$	0.011
$\text{XIIa}+\alpha_{2}\text{M}\to \text{XIIa}-\alpha_{2}\text{M}$	0.005
XIIa+AT-III→XIIa-AT-III	0.0013
XIIa+PAI-1→XIIa-PAI-1	0.96
XIIf+C1→XIIf-C1	0.185
$\text{XIIf}+\alpha_{2}\text{AP}\to \text{XIIf}-\alpha_{2}\text{AP}$	0.0091
XIIf+AT-III→XIIf-AT-III	0.0032
K+C1→K-C1	1
$\text{K}+\alpha_{2}\text{M}\to \text{K}-\alpha_{2}\text{M}$	0.29
K+PAI-1→K-PAI-1	3.6
K+AT-III→K-AT-III	0.0096
PCa+Va→PCa-Va	1200
PCa+VIIIa→PCa-VIIIa	1200
PCa+Va-Xa→PCa-Va-Xa	1200
PCa+VIIIa-IXa→PCa-VIIIa-IXa	1200
TM+IIa→IIa-TM	402
